# Inference of Coalescence Times and Variant Ages Using Convolutional Neural Networks

**DOI:** 10.1093/molbev/msad211

**Published:** 2023-09-20

**Authors:** Juba Nait Saada, Zoi Tsangalidou, Miriam Stricker, Pier Francesco Palamara

**Affiliations:** Department of Statistics, University of Oxford, Oxford, UK; Department of Statistics, University of Oxford, Oxford, UK; Department of Statistics, University of Oxford, Oxford, UK; Department of Statistics, University of Oxford, Oxford, UK; Wellcome Centre for Human Genetics, University of Oxford, Oxford, UK

**Keywords:** coalescence time, allele age, natural selection, heritability, machine learning

## Abstract

Accurate inference of the time to the most recent common ancestor (TMRCA) between pairs of individuals and of the age of genomic variants is key in several population genetic analyses. We developed a likelihood-free approach, called CoalNN, which uses a convolutional neural network to predict pairwise TMRCAs and allele ages from sequencing or SNP array data. CoalNN is trained through simulation and can be adapted to varying parameters, such as demographic history, using transfer learning. Across several simulated scenarios, CoalNN matched or outperformed the accuracy of model-based approaches for pairwise TMRCA and allele age prediction. We applied CoalNN to settings for which model-based approaches are under-developed and performed analyses to gain insights into the set of features it uses to perform TMRCA prediction. We next used CoalNN to analyze 2,504 samples from 26 populations in the 1,000 Genome Project data set, inferring the age of ∼80 million variants. We observed substantial variation across populations and for variants predicted to be pathogenic, reflecting heterogeneous demographic histories and the action of negative selection. We used CoalNN’s predicted allele ages to construct genome-wide annotations capturing the signature of past negative selection. We performed LD-score regression analysis of heritability using summary association statistics from 63 independent complex traits and diseases (average N=314k), observing increased annotation-specific effects on heritability compared to a previous allele age annotation. These results highlight the effectiveness of using likelihood-free, simulation-trained models to infer properties of gene genealogies in large genomic data sets.

## Introduction

The genomes of two individuals from a population are connected through genealogical relationships that lead to common ancestors. The distance, in generations, that separates these individuals and their common ancestor at a specific genomic location is referred to as time to most recent common ancestor (TMRCA), or coalescence time (Kingman 1982; Hudson 1983). Accurate prediction of pairwise coalescence times may be leveraged in several genomic analyses, such as detection of identical-by-descent (IBD) segments (Nait Saada et al. 2020) and the inference of the age of genomic variants (Albers and McVean 2020), which in turn can be utilized in the study of natural selection (Albrechtsen et al. 2010; Gusev et al. 2011; Hunter-Zinck and Clark 2015; Gazal et al. 2017; Palamara and Terhorst 2018).

Current approaches for the inference of TMRCAs rely on probabilistic modeling based on stochastic processes such as the coalescent with recombination (Hudson 1983; Wiuf and Hein 1999), and Markovian approximations of these processes (McVean and Cardin 2005; Marjoram and Wall 2006; Hobolth and Jensen 2014). Among these, coalescent hidden Markov models (Hobolth et al. 2007; Li and Durbin 2011; Sheehan et al. 2013; Schiffels and Durbin 2014; Terhorst et al. 2017; Palamara and Terhorst 2018) (or “coalescent HMMs,” reviewed in Spence et al. 2018) have been widely studied in recent years. Other recently developed methods enable inferring the ancestral recombination graph (ARG) for a set of individuals, which compactly represents the evolutionary history of a set of samples and includes their TMRCAs if branch lengths of the ARG are also estimated (Rasmussen et al. 2014; Speidel et al. 2019; Wohns et al. 2022; Zhang et al. 2023). Despite considerable progress in the development of probabilistic inference algorithms, likelihood-based inference for multi-locus data under the coalescent with recombination is often intractable (McVean and Cardin 2005). For this reason, available methods for TMRCA inference resort to simplifying assumptions that trade inference accuracy for computational efficiency, such as the discretization of TMRCA values within time intervals, or the use of approximate genealogical models (Li and Stephens 2003; McVean and Cardin 2005; Hobolth and Jensen 2014).

Difficulties in dealing with intractable likelihoods has motivated the development of “likelihood-free” inference strategies such as approximate Bayesian computation (ABC, Tavaré et al. 1997; Pritchard et al. 1999; Beaumont et al. 2002), where simulation is used to replace analytical modeling. These methods have been widely applied in population genetics and other fields (Estoup et al. 2004; Thornton and Andolfatto 2006; Becquet and Przeworski 2007; Fagundes et al. 2007; Patin et al. 2009; Toni et al. 2009; Beaumont 2010; Walker et al. 2010). Recent advances in probabilistic machine learning provided further momentum for the development of simulation-based inference (Cranmer et al. 2020), and simulation-based training of neural networks has been shown to offer advantages compared to approaches such as ABC (Chan et al. 2018; Korfmann et al. 2023). More broadly, deep learning algorithms have achieved state-of-the-art performance in several domains (Long et al. 2015; He et al. 2016, 2017; Vaswani et al. 2017; Devlin et al. 2018; Brown et al. 2020) and are now emerging as an effective tool in genomic applications. These include predicting functional effects of noncoding variants (Zhou and Troyanskaya 2015; Kelley et al. 2016, 2018; Zhou et al. 2018), basecalling of nanopore data (Teng et al. 2017), identifying the sequence specificities of DNA- and RNA-binding proteins (Alipanahi et al. 2015), inferring demographic history and population structure (Sheehan and Song 2016; Sanchez et al. 2021; Meisner and Albrechtsen 2022), inferring local ancestry (Montserrat et al. 2020) or geographic location (Battey et al. 2020), estimating mutation (Burger et al. 2022) and recombination rates (Adrion et al. 2020), detecting selective sweeps (Xue et al. 2021; Caldas et al. 2022; Hejase et al. 2022) and introgression (Gower et al. 2021), and generating synthetic data (Killoran et al. 2017; Sinai et al. 2017; Montserrat et al. 2019; Wang et al. 2021). However, although some preliminary work exists (Khomutov et al. 2021), the use of supervised learning approaches to infer genealogical relationships remains underexplored.

We developed an algorithm, called CoalNN, that uses a simulation-trained convolutional neural network (CNN) to jointly predict pairwise TMRCAs and recombination breakpoints, and further utilizes these predictions to estimate the age of genomic variants. While not requiring explicit probabilistic modeling, CoalNN achieves similar or improved inference accuracy compared to existing model-based methodology in a variety of simulated scenarios. CoalNN remains computationally efficient when applied to pairwise TMRCA inference, improving upon optimized coalescent HMMs in settings where GPU hardware is available. We use transfer learning to speed-up the training of CoalNN across different data types and evolutionary parameters, and perform interpretability analyses to gain insights into the combinations of genomic features used by the network to perform TMRCA prediction. We apply CoalNN to infer the age of ∼80 million variants identified in 26 populations from the 1,000 Genomes Project (1 kGP) (1000 Genomes Project Consortium 2015) and observe allele age variation consistent with population-specific demographic histories and the action of negative selection. Finally, we leverage CoalNN’s predicted allele ages to construct genome-wide annotations. Because evolutionary pressures affect the distribution of allele ages (Maruyama 1974; Kiezun et al. 2013), we use this annotation to capture the effects of natural selection along the genome (Gazal et al. 2017; Palamara and Terhorst 2018). We then test this annotation using stratified LD-score regression (S-LDSC Finucane et al. 2015; Gazal et al. 2017) and summary association statistics from 63 independent complex traits and diseases (average N=314k) to analyze trait heritability, detecting effects on heritability that complement existing evolutionary annotations.

## Materials and Methods

### Overview of the CoalNN Method

For a given pair of haploid individuals and a chromosomal region, we aim to predict a TMRCA value for each genomic site from a set of sites along the genome. To this end, CoalNN uses a convolutional neural network trained through realistic coalescent simulations, which is used to map the genomic data provided in input to target TMRCA values. The network receives in input the raw genotype values for each genomic variant, as well as a set of basic features. For each site, these include the minor allele frequency (MAF), physical and genetic distances (in base pairs [bp] and centimorgan [cM], respectively), and the number of consecutive identical-by-state (IBS) sites between the analyzed pair of individuals, in either direction.

**Fig. 1.**
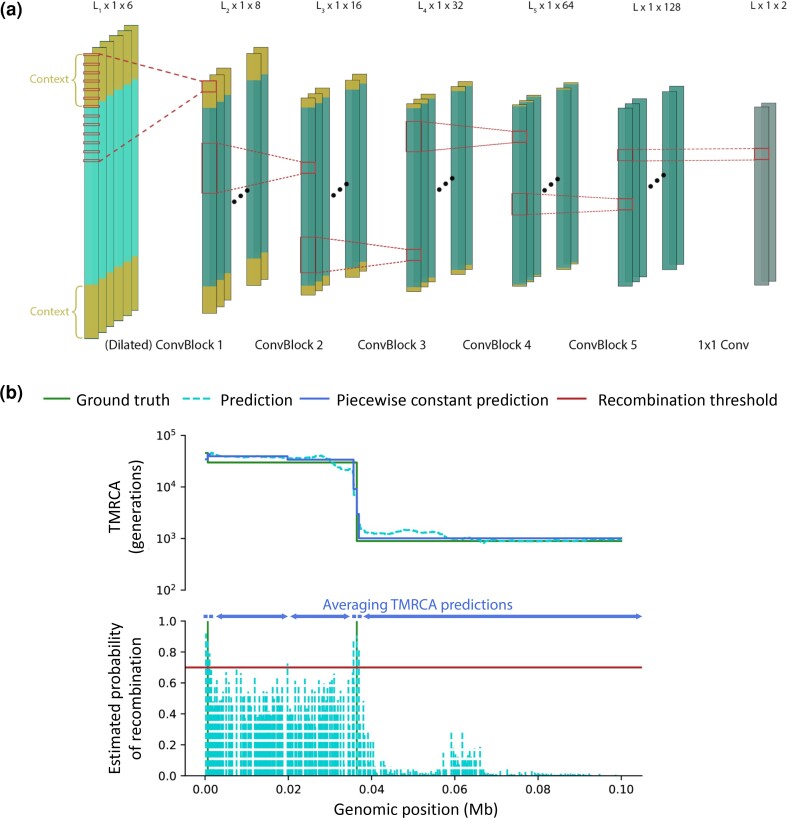
Overview of the CoalNN model. (*a*) CoalNN comprises a batch normalization layer followed by five convolution blocks (convolution layer + batch normalization + ReLU) and a final 1×1 convolution layer. The input sequence includes additional contextual data (denoted by ‘Context’ in the figure). The view offered here is simplified: in practice, a convolutional layer goes through all input channels and the outputs are summed to create one of the output channels. This process is repeated with a new convolutional layer for every output channel. (*b*) When making the output piecewise constant, CoalNN averages all inferred TMRCAs between consecutive genomic sites with an estimated probability of recombination that exceeds a user-specified threshold.

For a pair of haploid individuals, TMRCAs along the genome may be represented as a piecewise constant function taking positive real values, where each TMRCA interval is delimited by past recombination events. Therefore, in order to facilitate the output of piecewise constant TMRCA values, CoalNN jointly predicts the presence of a recombination breakpoint at each site. These estimated breakpoint positions are then utilized to produce piecewise constant TMRCA predictions, as shown in figure 1*b*. The overall network architecture, which comprises ∼130K trainable parameters, is illustrated in figure 1*a*.

**Fig. 2.**
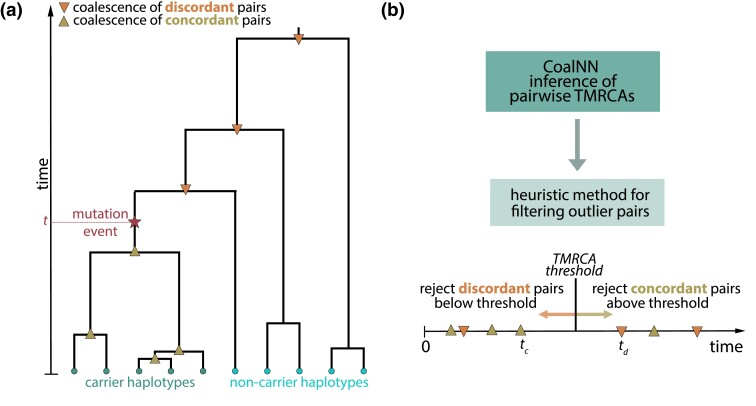
Procedure for dating genomic variants. (*a*) At a given genomic site, individuals are connected through underlying genealogical relationships. We aim to infer the time *t* at which a mutation arose (denoted by the star) and resulted in carrier haplotypes and noncarrier haplotypes. (*b*) When dating variants, CoalNN first infers TMRCAs across all concordant (two carriers) and discordant (one carrier and one noncarrier) pairs of haplotypes and then rejects outlier pairs using the heuristic approach developed in Albers and McVean (2020). The TMRCA rejection threshold is computed by minimizing the total number of rejected pairs. The predicted age estimate for the variant is obtained by averaging the maximum coalescence time across concordant pairs $t_{c}$ and the minimum coalescence time across discordant pairs $t_{d}$ after filtering.

Finally, CoalNN implements a strategy described in Albers and McVean (2020) to process the set of predicted pairwise coalescence times and further produce an estimate for the time of origin of each observed genomic variant (fig. 2*a*). In more detail, given a genomic variant, an age estimate is computed by averaging the maximum TMRCA across all concordant pairs (i.e., those for which the variant is carried by both haplotypes) and the minimum TMRCA across all discordant pairs (those for which the variant is carried by only one haplotype), as shown in figure 2*b*.

The simulation-based strategy used in CoalNN introduces additional computation during the training step, which is not required in approaches such as coalescent HMMs, but allows circumventing the need for complex probabilistic modeling. This facilitates inference in scenarios that are easy to simulate but difficult to model. For instance, inferring TMRCAs using CoalNN for input data where markers are nonrandomly ascertained, such as SNP array data, only requires simulating data under the ascertainment scheme. Coalescent HMMs, on the other hand, rely on a probabilistic modeling of the relationship between coalescence time and allele frequencies (Terhorst et al. 2017; Palamara and Terhorst 2018; Spence et al. 2018). Other desirable modeling features, such as the possibility to account for noncrossover gene conversion events (Wiuf and Hein 2000) can be effectively simulated but have not yet been incorporated into coalescent HMMs. To reduce computation during the training step, a CoalNN model trained in a given configuration may be adapted to work in related scenarios using transfer learning, by fine-tuning its parameters across each network layer.

### Neural Network Architecture

Since changes in the TMRCA value are linked to the presence of recombination breakpoints, CoalNN aims to jointly infer these target values using a multitask learning approach that relies on an underlying shared representation (Caruana 1997), hence allowing to exploit commonalities and differences across tasks (e.g., the presence of a recombination breakpoint is informative of a change in TMRCA value). We adopt the Huber loss function for the regression task of TMRCA prediction and the cross entropy loss for the classification task of breakpoint prediction. The Huber loss is equivalent to root mean squared error around zero and the mean absolute error otherwise, which provides increased robustness to outliers compared to the RMSE loss. We implemented the method introduced in Kendall et al. 2018 to simultaneously learn both objectives using homoscedastic uncertainty, so that the weights applied to each task in the loss function are also learnt. Our approach applies a convolutional neural network on genomic windows of fixed length *L*, with additional genomic data provided as context. The input data contain the AND and XOR functions applied to haplotype values for sequencing and SNP array data and raw imputation dosage values for imputed data. The network consists of multiple blocks comprised of batch normalization and convolution layers, followed by ReLU activation functions. Because the coalescent with recombination may be accurately approximated using Markov processes (McVean and Cardin 2005; Hobolth and Jensen 2014; Wilton et al. 2015), we expect the local connectivity of convolutional blocks to provide a suitable model for the TMRCA and recombination breakpoint prediction tasks. Convolution kernel sizes were chosen to allow a large receptive field in order to capture long identical-by-descent segments characterizing recent ancestry while maintaining a relatively low number of parameters for computational efficiency. The final network output contains for each of the *L* sites, the TMRCA prediction and the unscaled estimated probability of that site being a recombination breakpoint. Additional details on the network architecture are provided in the supplementary note, Supplementary Material online.

### Training Procedure and Simulation Parameters

Given a set of evolutionary parameters, such as demographic model and mutation rate, a data modality, such as sequencing, SNP array, or imputed data, and any additional parameters, such as phasing and genotyping error rates, we use simulation to generate synthetic genotype and genealogical data for training and validation. Because all samples from a simulation are related through underlying genealogical relationships, relying on a single simulation during training would lead to overfitting. To circumvent this issue, at the start of each training epoch CoalNN generates data from 64 independent coalescent simulations, each providing a single pair of individuals for the current training batch. If a nonconstant recombination rate is specified, each independent simulation also randomly samples a different genomic region, so that several subsets of the genetic map are observed during training.

For all validations and method comparisons, we simulated an input sequence of 30 cM for 150 individuals from a European [CEU (1000 Genomes Project Consortium 2015)] demographic model, using the genetic map inferred in Spence and Song (2019) and a constant mutation rate of $1.65\times 10^{-8}$ per base pair, per generation (Palamara et al. 2015). Root mean squared (RMSE) and mean absolute error (MAE) values for testing and comparing methods were calculated across all pairs and genomic sites for each approach, with random seeds not previously used during the training of CoalNN. When performing training and inference in sequencing data, we only retained variants that were polymorphic in 150 random individuals, so that the distance between consecutive variants remained approximately constant regardless of sample size. For SNP array data, polymorphic variants were subsampled to match the genotype density and allele frequency spectrum observed in the target data, which generally depends on the choice of array and analyzed samples. For these analyses, we trained models using the frequency spectrum observed in the UK Biobank (UKBB) data set (Bycroft et al. 2018). For imputed data, variants were first downsampled to create array-like data and then imputed using Beagle 5.1 (Browning et al. 2018) and a simulated diploid reference panel of size $n_{ref}$, where $n_{ref}$ was randomly chosen to be between 300 and 2,000. When deploying the model to analyze data from the 1,000 Genomes Project (1kGP, described below) we trained CoalNN through transfer learning with simulated sequencing data, using demographic models and genetic maps inferred for each population in Spence and Song (2019). We used the msprime simulator (v.1.0) (Kelleher et al. 2016) for all simulations and used the Adam optimizer with a learning rate of 0.001. We parallelized several steps of these procedures, such as the running of independent simulations, using multiple cores.

When comparing TMRCA estimates for CoalNN and ASMC (Palamara and Terhorst 2018), we used ASMC’s Python interface to precompute the decoding model used by ASMC and to run TMRCA inference. Calculation of ASMC’s decoding model requires a user to specify a time discretization for the HMM. To determine these intervals, we used quantiles of the coalescent distribution, as previously done (Terhorst et al. 2017; Palamara and Terhorst 2018). We used 200 quantiles as a default, but further tested the effect of the HMM discretization on ASMC’s accuracy and computation time requirements on sequencing data using 100, 200, 300, and 400 quantiles. For sequencing and imputed data, we set the decoding_mode parameter to sequence and did not use the conditional site frequency spectrum (CSFS) in the emission model, while for array data we set the decoding_mode to array and set the skip_CSFS_distance parameter to 0. ASMC does not support a floating point input representation of genotype data, so when analyzing imputed data with ASMC we binarized the input by rounding genotype dosages. Both CoalNN and ASMC were evaluated using simulations that used random seeds not used during the training of CoalNN.

### Sampling Procedure for Imbalanced Data

Because both recombination breakpoints along the sequence and very recent TMRCAs are observed in only a minority of data points during training, they create possible issues due to imbalanced data. To address the low frequency of recombination breakpoints, we use a weighted binary cross-entropy loss to predict recombination breakpoints, where the weights for the terms associated to recombination and no recombination in the loss are inversely proportional to the number of training elements in each class. In addition, we oversample recent TMRCAs by adopting a relatedness-informed sampling procedure during the construction of input batches. For a given training set simulation consisting of 2n haploid individuals, the first pair of haplotypes is randomly sampled. We then choose the next pair by uniformly sampling one of the haplotypes that have been previously processed and then selecting the haplotype with smallest average TMRCA (across the entire simulated chromosomal region) among the 2n−2 remaining samples. We repeat this procedure until all haplotypes have been paired. Although this approach increases the accuracy for recent TMRCA prediction, it introduces biases due to a deviation between observed and expected TMRCA distributions (see supplementary fig. 2, Supplementary Material online). To mitigate this issue, we alternate between uniform and relatedness-informed sampling at every other training epoch, with the training and validation losses shown in supplementary figure 3, Supplementary Material online, while saving the model weights corresponding to the minimum validation loss achieved during training.

To further address the scarcity of recent TMRCAs in the training data, we predict log-TMRCA rather than TMRCA values. This penalizes the ratio between ground truth and prediction, rather than their absolute distance, preventing the loss from being dominated by large TMRCA values. Finally, we designed a validation procedure that focuses on recent TMRCAs after each training epoch. To this end, we only used the top 5% closest pairs of haplotypes for validation by computing the average TMRCA across all sites for all pairs of samples, sorting the pairs in ascending order, and retaining the top 5%. The entire spectrum of possible TMRCA values is still represented in the validation set, but this process ensures that rare events such as recent coalescence are given more weight. We further adopted a weighted huber loss as validation score, which assigns more importance to under-represented recent and extremely old TMRCA regions of the genome.

### Transfer Learning

A trained CoalNN model may be adapted to varying parameters, such as demographic history, through transfer learning. For our experiments, we trained a baseline CoalNN model for a European [CEU (1000 Genomes Project Consortium 2015; Spence and Song 2019)] demographic model, and later fine-tuned all layers of this model to work in other settings, such as a demographic model of constant size ($N_{e}=10,000$) used for benchmarking and method comparison. Retraining CoalNN using an Nvidia A100 GPU card and 6 CPUs took approximately 30 and 5 h on sequencing and array data, respectively, while training new models took ∼40 and ∼20 h, respectively, with a substantial fraction (∼40%) of this time spent generating and processing the training data. Similarly, when analyzing the 1kGP data set, we loaded the weights of the baseline model and retrained CoalNN using population-specific demographic models and genetic maps.

### Beta-coalescent and Non-crossover Gene Conversion

One of the advantages of adopting the simulation-based training strategy used by CoalNN is that it can be applied to organisms whose evolutionary dynamics deviate from widely studied models, for which fewer or no likelihood-based approaches have been developed. This is the case, for instance, for the dynamics of several marine species (Hedgecock and Beaumont 1994; Hedrick 2005; Steinrücken et al. 2013) or viral spread (Menardo et al. 2021), where substantial variation in reproductive success may lead a small group of individuals to have many descendants. These processes have been modeled using a family of models that allow multiple merger events, called Λ-coalescents (Eldon and Wakeley 2006; Birkner and Blath 2008). Within this family, we focus on the Beta-coalescent (Schweinsberg 2003; Birkner et al. 2013), whose behavior is governed by a parameter α∈(1,2]. Small *α* values lead to many multiple merger events and a burst of recent coalescence, while values close to 2 lead to TMRCA distributions close to those of the Kingman coalescent. We performed experiments where CoalNN was trained on data simulated from the Beta-coalescent with parameters α=1.5, α=1.3, and α=1.1 for $N_{e}=50,000$ and α=1.8 for $N_{e}=20,000$. These $N_{e}$ values were selected in order to approximately match the TMRCA distribution and genetic variation observed in a Kingman coalescent with constant $N_{e}=10,000$.

We also explored a second scenario involving evolutionary features that are currently undermodeled in likelihood-based approaches, training CoalNN to predict TMRCAs in simulations that involve noncrossover gene conversion events (NCGC), in addition to crossover events. NCGC involves the transfer of genetic regions from one sequence to a second highly homologous sequence (Chen et al. 2007; Halldorsson et al. 2016). These regions may harbor polymorphic variants, leading to the presence of heterozygous sites within NCGC tracts, even when these tracts are flanked by regions where individuals share recent common ancestors (Palamara et al. 2015; Tian et al. 2019, 2022). If NCGC is not modeled during inference of TMRCAs, these heterozygous sites may be erroneously assumed to be the consequence of de-novo mutation events, leading to biased estimates. Although some parsimony-based algorithms for genealogical reconstruction have included heuristics aimed at capturing the effects of NCGC (Ignatieva et al. 2021), current coalescent HMM approaches do not model NCGC, in part owing to its non-Markovian nature (Wiuf and Hein 2000). We therefore performed simulations that include NCGC events, generated at a rate between $10^{-8}$ and $4\times 10^{-8}$, with gene conversion tract lengths of 300 basepairs (Palamara et al. 2015; Tian et al. 2019, 2022), with a constant crossover recombination rate of $10^{-8}$ for a population of constant effective population size $N_{e}=10,000$. Assuming that accurate estimates of NCGC rate and tract length are available, we trained a CoalNN model on simulations that include NCGC events in two steps. We first trained a baseline model using simulations that do not include NCGC and then used transfer learning to retrain this model with simulations that include NCGC events. For each NCGC rate, we compared the CoalNN model which was trained on data with NCGC to the baseline CoalNN model and ASMC, where NCGC is not modeled.

### Forming Piecewise Constant TMRCA Estimates

Ancestors shared along the genome by pairs of haploid individuals change as a result of recombination events, so that pairwise TMRCAs are expected to take the form of a piecewise constant function. However, raw CoalNN predictions tend to take different values at each site. CoalNN therefore implements a postprocessing step that leverages the recombination breakpoints predicted by the network to refine TMRCA predictions and allow producing piecewise constant estimates. In more detail, we average the TMRCA values inferred between genomic sites at which the estimated probability of observing a recombination event exceeds a specified threshold, as illustrated in figure 1*b*. We observed this approach to lead to accuracy improvements for both sequencing and array data, with optimal probability thresholds around 0.7 and 0.55 respectively. We determined these thresholds by performing a grid search, as detailed in supplementary figure 1*a*–*d*, Supplementary Material online.

### Dating of Genomic Variants

We used the pairwise coalescence times inferred by CoalNN to predict the time of origin (in generations before present) of genomic variants. For a given variant, the maximum TMRCA across all concordant pairs (those which are homozygous for the derived allele) provides a lower bound on the age of the variant, while the minimum TMRCA across all discordant pairs (those which are heterozygous) provides an upper bound (fig. 2*a*). However, due to noise in the TMRCA estimates, in practice the lower bound may be larger than the upper bound. To address this potential issue, we use the heuristic approach described by Albers and McVean (2020) and illustrated in figure 2*b* to filter out outlier TMRCAs, removing all concordant (resp. discordant) pairs above (resp. below) a TMRCA threshold. By increasing this TMRCA threshold, fewer concordant pairs and more discordant pairs will be rejected (and vice versa if decreasing the threshold). We select the threshold value that minimizes the total number of rejected pairs. The fraction of TMRCA estimates that are filtered out is provided in output and may be used as a proxy for the quality of the inferred variant age.

We compared allele age estimates for CoalNN, Relate (v1.1.6) (Speidel et al. 2019), and tsinfer+tsdate [tsinfer v0.2.1 (Kelleher et al. 2019) and tsdate v0.1.3 (Wohns et al. 2022) run successively]. Both Relate and tsinfer+ tsdate output allele age estimates as a range reflecting the lower and upper ends of the genealogical branch the mutation is estimated to be on. To obtain a point estimate for the age of an allele, we used the average of lower and upper ends of this range. CoalNN and Relate were provided with the simulated demographic model, while tsdate assumes a constant population size of 10,000 diploid individuals. All methods produced highly biased age estimates for singleton variants, for which no concordant pairs are available. We therefore excluded singletons from further analyses. We also considered GEVA (v1beta) (Albers and McVean 2020) in our benchmarks. However, GEVA produced estimates for ∼50% of nonsingletons variants; for these variants, we observed a lower accuracy compared to the other methods we considered, so we restricted our benchmarks to CoalNN, Relate, and tsinfer+tsdate.

### Interpretability Analyses

CoalNN’s input contains six basic genomic features, which are propagated through multiple ConvBlocks to compute predictions. In order to explore how the network relies on the input features to calculate TMRCAs, we obtained saliency maps (Simonyan et al. 2014; Zeiler and Fergus 2014) on sequencing data for simulated European samples. For a given pair of haplotypes and a given genomic region, we used the following procedure:

1. Perform a forward pass on the input of shape $L_{1}\times 1\times 6$, and obtain a TMRCA prediction per site of shape L×1×1. Multiply the predicted TMRCAs by $10^{6}$ to rescale for easier visualization and to avoid vanishing gradients (this is preferred to exponentiating as it does not affect the relative importance given to different input values).
2. Calculate the absolute value of the gradient (gradient norm) of the rescaled TMRCA on a target site with respect to the normalized input (i.e., the input after going through the first batch normalization layer), resulting in $6\times L_{1}$ gradient values. Note that the gradient is computed with respect to the normalized version of the input and not the raw input, so that all features are on the same standardized scale.
3. Multiply the gradient norm by the normalized input norm element-wise (known as the input×gradient method, Shrikumar et al. 2016) and visualize results on an $6\times L_{1}$ grid. Note that as we do not consider gradient directions, we only visualize absolute values in saliency maps.

In addition to using this procedure for producing and visualizing saliency maps, we further used the gradients to explore the relevance of the various input features in specific informative settings. Feature combinations for which we computed gradients (reporting average and SE across simulations) included heterozygous sites (XOR=1) with high MAF (>30%); rare (MAF<5%) homozygous (AND=1) variants; and regions of high (≥90th percentile) recombination rates. We often focused on sites where pairs of individuals have recent or deep coalescence times, which we defined as TMRCA<200 and TMRCA>100,000 generations, respectively.

We also performed perturbation analyses, in which we applied changes to specific inputs (while keeping all other inputs fixed) and measured variation in the network’s prediction. In more detail, given a pair of haplotypes, we either subtracted 0.05 from MAFs at all homozygous (AND=1) sites or added 0.05 to MAFs at all heterozygous (XOR=1) sites, and calculated the difference in TMRCA predictions before and after the perturbation. If the MAF value at a site was smaller (resp. higher) than 0.05 (resp. 0.45), we set the MAF to singleton frequency (resp. 0.5).

Finally, we tested whether the network learns to model standard features used in coalescent HMMs, such as the sharing of alleles between the two input haplotypes. To this end, we loaded the weights of the CoalNN model trained on imputed data, which takes raw genotypes as inputs rather than the XOR/AND functions, and applied it to sequencing data. To test whether the model automatically learns these features, we measured Pearson correlation between each of the eight channels in the output of the first ConvBlock and the XOR/AND functions. Note that the output of the first ConvBlock has shape ${\text{L}}_{2}\times 1\times 8$, while each logic function has shape ${\text{L}}_{1}\times 1\times 1$ (see fig. 1*a*). In order to compute correlations, we only considered the ${\text{L}}_{2}$ central sites in the functions (discarding $\frac{{\text{L}}_{1}-{\text{L}}_{2}}{2}$ sites on both ends), and we assumed a one-to-one mapping between the output of the ConvBlock and the logic gates. For all of these analyses, we averaged results across all genomic sites and pairs in a simulation.

### Computation of Standard Errors

Unless otherwise indicated, standard errors (SE) were computed over 10 independent simulations (using random seeds not seen in training) for analyses performed on simulated data. For real data analyses, we applied bootstrapping with 30 genomic regions as resampling units. These 30 genomic regions were obtained by dividing the autosomal chromosomes in contiguous regions with equal numbers of variants.

### 1,000 Genomes Project Data Set Preprocessing

We analyzed the GRCh38-build 1kGP Phase 3 data set (1000 Genomes Project Consortium 2015), which was lifted using CrossMap (version 0.5.4) (Zhao et al. 2014) from GRCh37 (1kGP release 20130502). The data set comprises 2,504 unrelated samples from 26 populations. To estimate allele ages, CoalNN uses knowledge of whether an allele is ancestral or derived. In order to recode the genotype data from reference/alternate to ancestral/derived, we used ancestral allele annotations provided with the data set. These were inferred from Ensembl multiple alignments (human assembly GRCh37) using Ortheus in the Enredo–Pecan–Ortheus (EPO) pipeline (Paten et al. 2008), resulting in either a high-confidence call, a low-confidence call, or no call for each variant. We used these annotations to enforce ancestral/derived encoding for all variants for which a call was available. We retrained CoalNN on each of the 26 groups by transfer learning, using population-specific demographic models and GRCh38 recombination maps (Spence and Song 2019). This resulted in 26 trained CoalNN models, one per 1kGP population.

### Allele Age Estimation in the 1,000 Genomes Project Data Set

We inferred allele ages for all polymorphic variants identified in the 1kGP data set within each population separately, using CoalNN weights trained without NCGC. We aggregated age estimates for the following five super-groups (see supplementary table 1, Supplementary Material online), using population assignments provided with the 1kGP data set: African (AFR), American (AMR), East Asian (EAS), European (EUR), and South Asian (SAS). For each super-group and each variant, we computed a weighted average of the lower age estimates across all constituent populations. We used the number of concordant pairs retained after filtering as weights for each population’s contribution to the average. Similarly, we computed a weighted average of the upper age estimates using the number of discordant pairs after filtering as weights. Age estimates were then obtained by taking the mean of these upper and lower weighted averages. We dated ∼42 million variants in AFR, ∼28 million in AMR, ∼24 million in EAS, ∼24 million in EUR, ∼27 million in SAS, and ∼80 million overall. When performing S-LDSC analyses (described below), we also combined age estimates from all 26 populations, using the same procedure. We have made all age estimates publicly available (see URLs). Unless otherwise specified, we only retained variants with high-confidence ancestral state annotations and excluded singleton variants in all downstream analyses.

### Variant Effect Predictor Annotation

We extracted filtered pathogenicity annotations from the Ensembl Variant Effect Predictor (VEP) (McLaren et al. 2016) generated by SIFT (Sim et al. 2012) (annotated as “deleterious” or “tolerated”) and by PolyPhen-2 (Adzhubei et al. 2010) (annotated as “possibly damaging,” “probably damaging” or “benign”) for all analyzed variants, and lifted their coordinates from GRCh37 to GRCh38. After excluding variants with low-confidence or missing ancestral state, we obtained 115,288 variants in AFR, 77,672 in AMR, 70,049 in EAS, 80,782 in EUR, 48,358 in SAS for Polyphen-2, and 138,573 variants in AFR, 93,034 in AMR, 84,523 in EAS, 97,753 in EUR, 57,605 in SAS for SIFT. We computed the cumulative distribution functions of each annotated group of variants in each super-group, stratified by derived allele frequency.

### S-LDSC Analyses

We built annotations to perform stratified LD-score regression analysis (Finucane et al. 2015), using the procedure described by Gazal et al. (2017) to analyze the effect of an annotation based on allele ages on complex trait heritability. LD-score regression relies on the principle that summary association statistics from a genome-wide association study (GWAS) for a polygenic trait will tend to be larger for genomic markers that are in high LD with several other variants (i.e., have a high “LD-score”) compared to those in low LD with other variants (having a low LD-score) (Bulik-Sullivan et al. 2015). Stratified LD-score regression (Finucane et al. 2015) aims to estimate the contribution of variants that are found within specific genomic annotations to the heritability of a trait for which GWAS summary statistics are available. These genomic annotations can be binary (e.g., whether variants are in an intronic region) or continuous (e.g., the rate of recombination). Several continuous annotations related to evolutionary properties have been estimated to have a significant effect on human complex trait heritability (Gazal et al. 2017; Palamara and Terhorst 2018). These have included an annotation encoding MAF-adjusted allele ages along the genome (Rasmussen et al. 2014), which likely captures the action of negative selection on variants linked to the analyzed complex traits and diseases (Maruyama 1974; Kiezun et al. 2013).

We constructed 26 different MAF-adjusted annotations using allele age predictions obtained by CoalNN for each 1kGP population. We partitioned variants using the same 10 MAF bins (all with MAF≥0.05) used by Gazal et al. (2017); within each MAF bin, we quantile normalized the allele ages to a standard normal distribution. We used the same approach to construct six additional MAF-adjusted and quantile-normalized annotations: one for each population super-group and one encompassing all 26 populations. Overall, we produced 32 allele age annotations and we evaluated them by applying S-LDSC to summary association statistics from 63 independent diseases and complex traits, described in supplementary table 2, Supplementary Material online. S-LDSC analyses were performed using the S-LDSC software (Finucane et al. 2015) and the baselineLD v2.2 model.

## Results

### Simulation Results

**Fig. 3.**
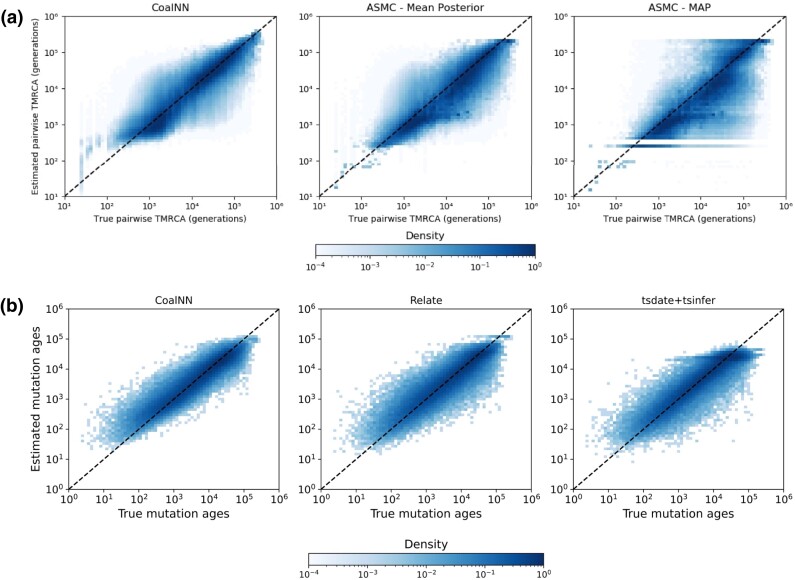
Pairwise TMRCA and allele age prediction on sequencing data. (*a*) True pairwise TMRCAs (*x* axis) versus those estimated by CoalNN and ASMC (*y* axis) under a European demographic model for one simulation. For TMRCA prediction performance of CoalNN and ASMC by decile of the true TMRCA distribution, see supplementary table 3, Supplementary Material online. (*b*) True nonsingleton variant ages (*x* axis) versus those estimated by CoalNN, Relate, and tsdate+tsinfer (*y* axis) under a constant diploid population size $N_{e}=10,000$.

We assessed the accuracy of CoalNN in inferring locus-specific pairwise TMRCAs and allele ages through extensive simulations of sequencing and SNP array data and further considered various scenarios of model misspecification, such as introducing phasing and genotyping errors, or using imputed data. We compared the pairwise TMRCA estimates inferred by CoalNN’s likelihood-free approach to both the MAP and the posterior mean estimates provided by ASMC [v.1.2 (Palamara and Terhorst 2018)], a coalescent HMM. CoalNN performed comparably to ASMC in sequencing and array data (see table 1, fig. 3*a*, and supplementary fig. 4*a*, Supplementary Material online), obtaining a similar root mean squared error (RMSE) and a slightly improved mean absolute error (MAE improvement of 2.33% (SE=0.24) and 2.71% (SE=0.32) for sequencing and array data, respectively). In these experiments, we observed slightly improved performance by CoalNN for more recent times, which was likely due to the oversampling of recent TMRCAs during training. As ASMC’s accuracy may be affected by a user-specified time discretization, we also tested ASMC under several discretization conditions, which however did not improve the results and led to higher computing time (see supplementary table 4, Supplementary Material online). We also performed experiments in which we provided raw sequencing data in input to CoalNN, rather than the precomputed AND and XOR features, using a constant demographic model with $N_{e}=10$ K. This resulted in a marginally decreased performance and a significantly increased time to convergence; we obtained a MAE of 6,238 (SE=28), RMSE of 11,356 (SE=67) for the model using raw genotypes compared to 6,098 (SE=29), and 11,278 (SE=56) for the model using the AND and XOR features. Training required 120 epochs using raw data, compared to 24 when using AND and XOR.

**Table 1.** Accuracy of Pairwise TMRCA Prediction.

		CoalNN	ASMC Mean Posterior	ASMC MAP
Sequencing	MAE	8,393 (35)	8,594 (30)	9,758 (45)
	RMSE	17,697 (97)	17,974 (120)	21,918 (126)
Array	MAE	15,936 (77)	16,382 (106)	28,352 (118)
	RMSE	29,679 (226)	29,569 (279)	57,131 (206)

Note.—We report the average performance in generations of CoalNN and ASMC for the mean absolute error (MAE) and the root mean squared error (RMSE) under a European demographic history model (CEU) across 10 simulations. Numbers in round brackets represent standard errors.

We also tested CoalNN’s accuracy in estimating allele ages using its TMRCA predictions (see Methods). We simulated data from a European demographic model and from a constant population size of $N_{e}=10,000$ and compared to allele age predictions obtained using Relate (Speidel et al. 2019) and tsinfer+tsdate (Kelleher et al. 2019; Wohns et al. 2022). In these simulations, CoalNN achieved the highest allele age estimation accuracy across several metrics, shown in figure 3*b*, supplementary figure 4*b*, Supplementary Material online and table 2.

**Table 2.** Accuracy of Estimated Allele Ages.

		RMSE	MAE	MAD	$r^{2}$
CEU	Relate	24,753 (215)	12,355 (93)	4,271 (46)	0.527 (0.002)
	tsinfer+tsdate	29,685 (280)	14,363 (118)	4,695 (48)	0.368 (0.003)
	CoalNN	23,325 (154)	11,689 (79)	4,001 (43)	0.582 (0.002)
Constant	Relate	11,029 (117)	5,341 (44)	1,647 (12)	0.632 (0.003)
	tsinfer+tsdate	12,909 (258)	5,864 (68)	1,557 (12)	0.526 (0.005)
	CoalNN	10,901 (125)	5,108 (46)	1,487 (11)	0.648 (0.002)

Note:—We report the average performance (in units of generations) of Relate, tsinfer+tsdate, and CoalNN on nonsingleton variants under a European demographic history model (CEU) and a constant population size ($N_{e}=10,000$) model across 10 simulations, with numbers in round brackets representing standard errors. We report the root mean squared error (RMSE), the mean absolute error (MAE), the median absolute deviation (MAD), and the square of the Pearson correlation coefficient ($r^{2}$).

Next, we validated the robustness of CoalNN to various types of model misspecification, including phasing errors, genotyping errors, an inaccurate demographic model and using imputed (rather than sequencing) data. We first measured the robustness of CoalNN and ASMC to the presence of genotyping and phasing errors, using simulated sequencing data under a European demographic model. In these simulations, CoalNN was more robust than ASMC across a range of simulated error rates, with the performance gap increasing for larger error rates (supplementary fig. 5, Supplementary Material online). For a switch (resp. genotyping) error rate of 0.1%, CoalNN had a 3.01% (SE=0.22) lower mean absolute error (MAE, resp. 2.97%, SE=0.22) compared to ASMC’s posterior mean estimates and a 14.22% (SE=0.23) lower MAE error (resp. 14.47%, SE=0.24) compared to ASMC’s MAP estimates; these MAE improvements increased approximately linearly with larger error rates. We tested CoalNN’s robustness to demographic model misspecification by simulating data under a constant population size of 10,000 diploid individuals, using model parameters trained on a European demographic model. For comparison, we also ran ASMC while incorrectly assuming a European demographic model. CoalNN generalized similarly to ASMC on sequencing data (2.84%, SE=0.19 MAE performance improvement over the ASMC mean posterior estimates compared to 2.32%, SE=0.24 MAE performance improvement when using the correct demography). ASMC, however, was more robust when using array data (−12.45%, SE=0.64 compared to 2.71%, SE=0.32). Finally, we simulated imputed data with reference panels of varying sizes and performed inference by rounding dosages and using the sequencing model weights. CoalNN’s performance did not significantly differ from ASMC’s across all reference panel sizes, as shown in supplementary table 5, Supplementary Material online.

We next aimed to apply CoalNN in settings for which likelihood-based inference approaches have been less studied, such as in simulations involving noncrossover gene conversion events (NCGC) or the Beta-coalescent process. NCGC events are known to occur in humans and other species (Williams et al. 2015; Halldorsson et al. 2016) but are not modeled in coalescent HMMs, also due to their non-Markovian nature (Wiuf and Hein 2000). During NCGC, polymorphic variants may be introduced within genomic regions that are shared by groups of individuals (Palamara et al. 2015; Tian et al. 2019, 2022). If not modeled, the presence of these variants introduced by NCGC may lead to biases in TMRCA estimation. We performed simulations that include different rates of NCGC (see Materials and Methods). We observed the TMRCA prediction error of CoalNN and ASMC to grow with the rate of simulated NCGC events (see supplementary table 6, Supplementary Material online). Although ASMC does not model NCGC, it may be possible to improve its performance in this setting, for example, by modifying its emission model to account for an estimated rate and length of NCGC tracts, which we did not explore. Using transfer learning to retrain a CoalNN model with simulations that include NCGC events, on the other hand, was sufficient to achieve higher robustness to the presence of NCGC events, significantly improving upon the performance of ASMC, and a CoalNN model trained only assuming crossover events. We also performed simulations under a Beta-coalescent model, which allows accounting for multiple merger events that are observed in organisms such as some marine species or viral evolution (see Materials and Methods, Hedgecock and Beaumont 1994; Schweinsberg 2003; Hedrick 2005; Birkner et al. 2013; Steinrücken et al. 2013; Menardo et al. 2021). Again, it may be possible to improve the performance of ASMC in this setting, for example, by mapping parameters of the Beta-coalescent process to demographic models that better capture the pairwise coalescence rate of a given set of parameters, which we did not explore. Training CoalNN using Beta-coalescent simulations, on the other hand, yielded good correlation between predicted and true TMRCAs, with CoalNN capturing clusters of recent coalescence observed under this model (see Materials and Methods, supplementary fig. 6, Supplementary Material online). Smaller alpha values resulted in more extreme multiple mergers; for α=1.1, in particular, CoalNN predictions overestimated low TMRCA values.

**Fig. 4.**
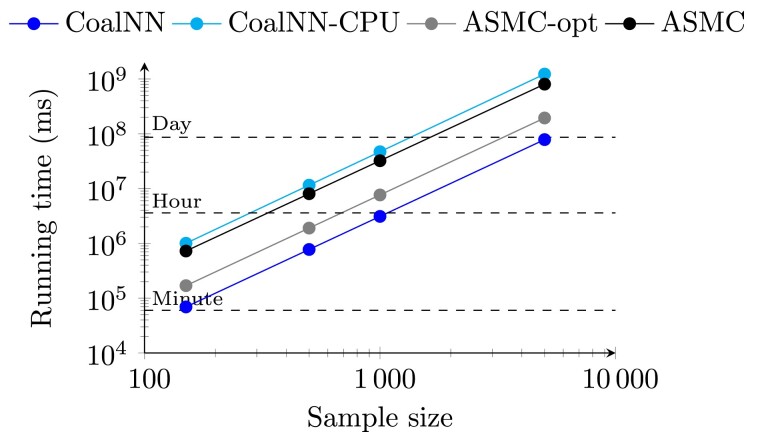
Running time evaluation. Running time (in milliseconds) of CoalNN (on a single A100 GPU card and a single CPU, and on a single CPU only) and ASMC (on a single CPU, optimized and nonoptimized version) on array data using the first 30 Mbp of chromosome 2 across 6,749 SNPs. The batch size for both methods is 64.

Finally, we evaluated the computational efficiency of CoalNN, comparing it with ASMC in a setting where both models have been previously tuned for a given set of parameters and are deployed to perform TMRCA inference. When inferring TMRCAs for random pairs of individuals from a data set, a trained CoalNN model run on a NVIDIA A100 GPU was ∼10.5× faster than ASMC run on an Intel Skylake 2.6 GHz CPU, as shown in figure 4. ASMC may be run in batch-optimized mode, where memory locality and single instruction/multiple data (SIMD) processing are leveraged for faster analysis of several pairs of contiguous individuals from the input genotype matrix. Under the same hardware configuration, CoalNN was ∼2.5× faster than batch-optimized ASMC. When run on a CPU architecture, CoalNN was ∼15.6× slower compared to its GPU performance and ∼1.5× slower than ASMC when applied to TMRCA inference in random individual pairs. Training CoalNN on a CEU demographic model required ∼52 h for sequencing data, ∼23 h for imputed data, and ∼9 h for SNP array data, using an Nvidia A100 GPU card and 6 CPUs. We used transfer learning, where a previously trained CoalNN model is fine-tuned for a new setting (e.g., a different demographic parameters, see Materials and Methods), to increase training speed; compared to the training of randomly initialized models, this approach was ∼1.5× faster for sequencing data and ∼5× faster for array data.

### Interpretation of CoalNN’s Predictions

While being accurate, CoalNN’s trained model may be hard to interpret, as it automatically learns a map between input data and inferred TMRCA values rather than relying on an explicitly designed model. We therefore investigated whether CoalNN implicitly infers and leverages genomic features that have been described and utilized in other probabilistic approaches, such as coalescent HMMs.

To this end, we first examined saliency maps, which use the gradient of the output with respect to the input as a way of quantifying the importance of different input regions in forming the model’s prediction (see Materials and Methods and supplementary fig. 7, Supplementary Material online for two representative examples). Examining regions where pairs of individuals shared recent common ancestors (TMRCA<200), we observed that the XOR feature, which reflects whether individuals are identical-by-state and is a standard input of coalescent HMMs (Li and Durbin 2011), remained informative for long stretches of the input region, indicating the presence of long shared haplotypes. Gradients for the AND feature, which allows quantifying whether the individuals are both carriers of a derived allele and was adopted in more recent coalescent HMMs (Terhorst et al. 2017), were more localized around the focal site. Larger gradients for the genetic distance feature, which is informative for the detection of recombination breakpoints, tended to be localized in regions of high recombination rates. For instance, when examining regions where TMRCA values are large (>100,000 generations), we observed an average gradient of 46.2 (SE=0.03) for regions with recombination rate in the 90th percentile or above, compared to 16.26 (SE=0.02) for lower recombination rates.

Next, we used input perturbations to test whether CoalNN learns to utilize more complex combinations of these basic features, such as MAF and allele sharing, that require complex probabilistic modeling in coalescent HMMs (Terhorst et al. 2017). Assuming no recurrent mutation, individuals sharing rare alleles are expected to coinherit these alleles from a recent common ancestor, since rare variants are on average younger than higher frequency variants (Kimura and Ohta 1973; Griffiths and Tavaré 1999). Similarly, individuals that are heterozygous for high frequency variants are more likely to share a distant common ancestor. To verify that CoalNN learns to rely on combinations of MAF and allele sharing to form its predictions, we perturbed MAF values for individuals based on their allele sharing. We observed that increasing the input MAF value by 5% at sites for which individuals are heterozygous for high frequency variants (XOR=1, MAF>45%) resulted in an average increase in predicted TMRCA of 181.5 (SE=3.7) generations, while decreasing the MAF at homozygous sites (AND=1, MAF<5%) resulted in an average decrease of predicted TMRCA of 27.6 (SE=1.7) generations (see Materials and Methods, supplementary fig. 8, Supplementary Material online).

Finally, we sought to verify that CoalNN would allow independently recovering basic engineered features, such as the XOR and AND logic functions, using raw haplotype data. To this end, rather than providing XOR and AND features in input to increase training efficiency, we provided CoalNN with raw genotype data, and observed that the output channels of the first hidden layer were significantly correlated with the XOR and AND logic functions (r=−0.172, SE=0.001 and r=−0.272, SE=0.001, respectively, see supplementary table 7, Supplementary Material online).

### Allele Age Prediction in 1,000 Genomes Project Populations

We applied CoalNN to 2,504 samples from the 1kGP Phase 3 data set and we inferred allele ages for ∼80 million variants (see Materials and Methods). We analyzed each of the 26 populations separately and aggregated estimates for each population group (labeled as AFR, AMR, EAS, EUR, and SAS, see Methods, supplementary table 1, Supplementary Material online). Allele ages inferred using CoalNN were highly correlated with previously published age estimates. For variants with high-confidence ancestral states, the average correlation across all 26 populations was r=0.3 (SE=0.07) for ages predicted by GEVA (Albers and McVean 2020), and r=0.67 (SE=0.11) for ages inferred by Relate (Speidel et al. 2019).

**Fig. 5.**
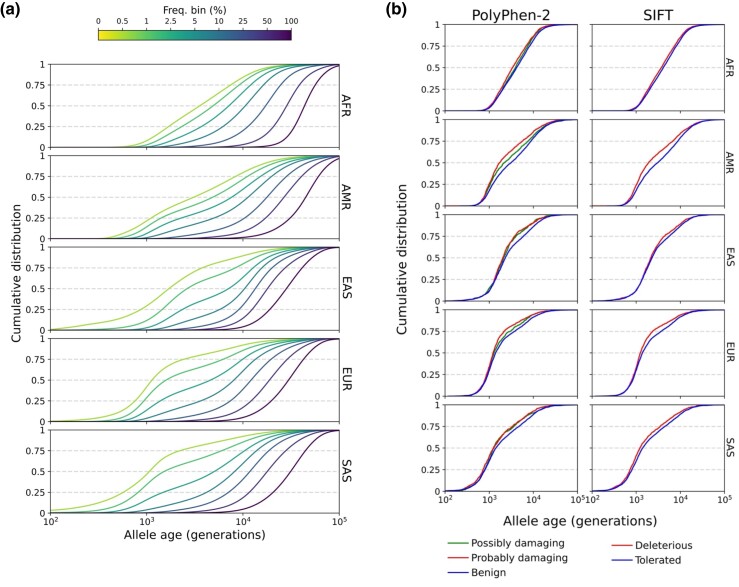
Age distribution of dated variants among different population groups. (*a*) Cumulative age distribution function of all dated variants across the human genome per population group. For each line, only nonsingleton polymorphic variants present in that population within a given derived allele frequency bin were considered. (*b*) Differences in allele age distribution between pathogenic mutations (annotated as such by PolyPhen-2 and by SIFT) and neutral variants for a derived allele frequency between 1% and 2.5% within each population group.

We analyzed the genome-wide distribution of frequency-stratified allele ages (see fig. 5*a*) and observed significant differences between populations, reflecting population-specific histories of migration and population size variation (1000 Genomes Project Consortium 2015; Albers and McVean 2020). For instance, for variants with derived allele frequency between 1% and 2.5% we observed a median age of 4,904 generations (SE=53) in AFR; 4,852 generations (SE=64) in AMR; 2,634 generations (SE=27) in EAS; 1,593 generations (SE=20) in EUR; and 1,517 generations (SE=29) in SAS (see supplementary table 8, Supplementary Material online). We replicated this experiment using CoalNN weights trained by assuming a constant population size of $N_{e}=20,000$ for three representative populations (CEU, CHS, YRI). Using this approach, which is likely to result in age estimates that are slightly less accurate (see supplementary fig. 9, Supplementary Material online), we observed similar patterns of allele age variation (see supplementary fig. 10 and table 9, Supplementary Material online), suggesting that the observed variation is not only due to the use of CoalNN models trained on population-specific demographic priors.

Next, we analyzed variation in the age distribution of frequency-stratified alleles predicted to have different pathogenic effects using SIFT (Sim et al. 2012) and PolyPhen-2 (Adzhubei et al. 2010), reflecting the action of negative selection (Albers and McVean 2020) (see Materials and Methods). To control for the relationship between allele frequency and allele age, we compared sets of neutral and potential pathogenic variants observed to have the same derived allele frequency (DAF). Allele age distributions of rare variants (1%<DAF<2.5%) annotated by PolyPhen-2 and SIFT are shown in figure 5*b*. Results for variants at other frequencies are illustrated in supplementary figs. 11 and 12, Supplementary Material online and summarized in supplementary tables 10 and 11, Supplementary Material online. We observed deleterious alleles to be younger than neutral alleles of the same frequency in every population group, consistent with the action of negative selection (Kiezun et al. 2013; Albers and McVean 2020) (e.g., median age of 900 generations, SE=19, for AFR for DAF<1% variants identified as deleterious by SIFT, compared to 1,061 generations, SE=23, for variants annotated as tolerated).

### Stratified LD-Score Regression Analysis

Stratified LD-score regression [S-LDSC (Finucane et al. 2015)] has been used to test whether genome-wide annotations built using evolutionary features are predictive of heritability enrichments across complex traits and diseases (Gazal et al. 2017). In particular, an annotation based on allele ages inferred using the ARGweaver algorithm (Rasmussen et al. 2014) has been observed to have significant effects on complex trait heritability (Gazal et al. 2017), likely reflecting the effects of natural selection on allele age variation. These observed enrichments persisted when conditioning on the per-allele effects of several other evolutionary annotations, including nucleotide diversity, a background selection statistic (McVicker et al. 2009) (*B*-statistic), average pairwise TMRCA estimated using ASMC (Palamara and Terhorst 2018) (${\text{ASMC}}_{\text{avg}}$), recombination rate, the level of LD in African populations (Gazal et al. 2017) (LLD-AFR), and CpG content (Zhang et al. 2021).

**Fig. 6.**
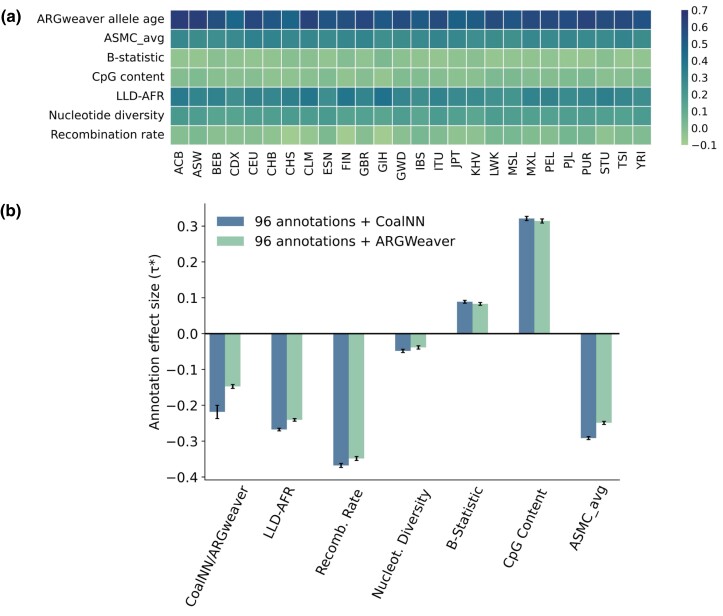
S-LDSC analysis of CoalNN MAF-adjusted allele age annotations. (*a*) We report correlations computed on common SNPs (MAF≥5%) between each of the 26 population specific MAF-adjusted CoalNN annotations and evolutionary annotations from the baseline model. ARGweaver allele age, ${\text{ASMC}}_{\text{avg}}$, and LLD-AFR annotations are also adjusted for MAF. Numerical results are reported in supplementary table 13, Supplementary Material online. (*b*) Effect size $\tau^{*}$ estimates (meta-analyzed across 63 independent diseases and complex traits listed in supplementary table 2, Supplementary Material online) of CoalNN MAF-adjusted allele age annotation on all 26 populations and of ARGweaver MAF-adjusted allele age annotation (Rasmussen et al. 2014), in marginal S-LDSC analysis conditioned on 96 baseline annotations (the full baseline model except for ARGweaver) (Gazal et al. 2017). We also report effect sizes of baselineLD evolutionary annotations [level of LD measured in African populations LLD-AFR, recombination rate, nucleotide diversity, *B*-statistic (McVicker et al. 2009), CpG content (Zhang et al. 2021), and average pairwise TMRCA ${\mathrm{ASMC}}_{\mathrm{avg}}$ (Palamara and Terhorst 2018)] after the introduction of either the CoalNN or ARGWeaver allele age annotation. Error bars represent standard errors of the meta-analyzed $\tau^{*}$ estimates. See supplementary table 14, Supplementary Material online for numerical results.

In order to test the informativeness of an annotation built using allele age estimates obtained through CoalNN, we constructed 26 MAF-adjusted annotations, one for each 1kGP population (see Materials and Methods). We found these annotations to be highly correlated with other MAF-adjusted evolutionary annotations present in the Baseline-LD model (Gazal et al. 2017) (see fig. 6*a*), including r=0.56, SE=0.01 (average across populations) for the ARGweaver allele age annotation; r=0.3, SE=0.01 for LLD-AFR, r=0.28, SE=0.004 for ${\text{ASMC}}_{\text{avg}}$; and r=0.16, SE=0.004 for nucleotide diversity. We tested the CoalNN allele age annotations by applying S-LDSC to summary statistics from 63 independent complex traits and diseases (see supplementary table 2, Supplementary Material online). We quantified the informativeness of an annotation by performing a meta-analysis of the heritability effect $\tau^{*}$ across these traits. The heritability effect $\tau^{*}$ is defined as the proportionate change in per-SNP heritability associated with a 1 s.d. increase in the value of the annotation, conditional on other annotations included in the baseline LD model (Gazal et al. 2017).

We first investigated the informativeness of the CoalNN annotations when conditioned on the full set of 97 annotations within the baseline-LD model, including the ARGweaver allele age annotation. In these conditional analyses, we observed the CoalNN annotations for several populations to have a significant effect (supplementary fig. 13*b*, Supplementary Material online). However, these effects were heterogeneous and occasionally in opposite directions (e.g., $\tau_{\text{ACB}}^{*}=-0.12$, SE=0.02, and $\tau_{\text{CHS}}^{*}=0.11$, SE=0.02). This heterogeneity may be linked to the high correlation between the ARGweaver and CoalNN MAF-adjusted allele age annotations (see supplementary fig. 13*a*, Supplementary Material online). Repeating these analyses after removing the ARGweaver annotation from the baseline model resulted in consistent effect directionality across populations (see supplementary fig. 13*c*, Supplementary Material online). We thus opted to evaluate the informativeness of the CoalNN annotations using analyses where the ARGweaver annotation is first removed from the baseline model, conditioning on the remaining set of 96 functional and evolutionary annotations.

Next, we aggregated allele age estimates across all 26 populations to obtain a single CoalNN annotation (see Materials and Methods). We assessed its heritability effect when no other evolutionary annotation is included in the model, by only conditioning on the 90 remaining annotations within the Baseline-LD model. In this scenario, the CoalNN annotation obtained a meta-analyzed effect size $\tau^{*}$ of −1.01 (SE=0.02), while performing the same analysis with the ARGweaver annotation resulted in a $\tau^{*}$ of −0.88 (SE=0.02). We repeated this analysis, this time conditioning on other evolutionary annotations (LLD-AFR, recombination rate, nucleotide diversity, B-statistic, CpG content, and ${\text{ASMC}}_{\text{avg}}$), observing a $\tau^{*}$ of −0.22 (SE=0.02) for CoalNN and of −0.15 (SE=0.01) for ARGweaver (fig. 6*b*, see supplementary fig. 14, Supplementary Material online for individual trait results). We observed larger effect sizes for evolutionary annotations when including ages inferred by CoalNN as a new annotation compared to when including ages predicted by ARGweaver, suggesting a larger amount of overlapping information between ARGWeaver and the other evolutionary annotations. Finally, we repeated these conditional analyses using population-specific annotations, observing heterogeneous but compatible effect sizes across groups (supplementary fig. 15, Supplementary Material online).

Overall, these results suggest that the CoalNN annotation captures heritability effects that are not captured by the ARGWeaver annotation or other evolutionary annotations contained in the baseline-LD model. The negative sign of the heritability effect size indicates that, after conditioning on allele frequency, alleles with a younger estimated age correspond to larger phenotypic effects, consistent with the action of negative selection (Kiezun et al. 2013; Gazal et al. 2017).

## Discussion

We developed CoalNN, a likelihood-free method that uses a convolutional neural network to predict pairwise coalescence times and recombination breakpoints from sequencing and array genotype data. Using extensive simulations, we found that CoalNN matches or improves upon the accuracy of current approaches for the inference of TMRCAs and the dating of genomic variants, while not requiring explicit probabilistic modeling or time discretization, and remaining computationally efficient. We applied CoalNN to the 1kGP data set and estimated the age of ∼80 million variants across 26 human populations. We observed differences in allele age distributions between populations, reflecting diverse demographic histories, as well as between predicted pathogenic and neutral alleles, reflecting the action of natural selection. We built genome-wide allele age annotations to capture the effects of selection and used stratified LD-score regression to analyze the genetic architecture of 63 diseases and complex traits. Using this approach, we showed that annotations built using CoalNN capture significant heritability effects, which are consistent with the action of negative selection on variants linked to these traits (Gazal et al. 2017; Palamara and Terhorst 2018). These effects were larger than those of a previous annotation based on allele ages and remained significant after conditioning on several other evolutionary annotations.

Overall, these results demonstrate that deep learning algorithms trained using simulation provide an effective route to inferring genealogical relationships for a set of sequenced or genotyped samples. More generally, simulation-based training enables circumventing difficulties linked to intractable likelihood calculations, providing an alternative to other likelihood-free strategies or inference under more approximate models. For instance, CoalNN could be easily adapted to models that include noncrossover gene conversion events, for which coalescent HMM models have not been developed, and for the Beta-coalescent process, for which few models exist [also see Korfmann et al. (2022) for recent work in this area]. CoalNN also achieved increased computational speed in TMRCA inference compared to ASMC in settings where GPU hardware is available. Although this work has focused on the inference of pairwise genealogical relationships and allele ages, CoalNN could also be used as a building block within other algorithms that infer the full genealogy for larger sets of samples. Pairwise coalescence times inferred using CoalNN may be used to sequentially thread new samples in an existing genealogy, as recently done in the ARG-Needle algorithm (Zhang et al. 2023) using ASMC (Palamara and Terhorst 2018). Similarly, improved allele age estimates could be utilized to more accurately infer the age of ancestors used to assemble genealogies in the tsinfer algorithm (Kelleher et al. 2019), as done in the tsdate approach (Wohns et al. 2022).

We note a few areas of future improvement, as well as limitations of this work. Although a trained model may also be applied to data using standard CPU hardware, CoalNN relies on GPU hardware for optimal computational performance. Moreover, although we observed that CoalNN trained on a constant demographic prior performs well under different scenarios, properly generalizing to varying evolutionary settings, including demographic models, mutation and recombination rates, requires additional training and therefore computational resources. Extending CoalNN so that it can be more efficiently adapted to varying evolutionary parameters, or to allow it to directly learn these parameters, is a desirable area of future improvement. For instance, it may be possible to use CoalNN’s estimated recombination probabilities to estimate local recombination rates. We performed an exploratory experiment, in which we trained CoalNN without using genetic maps (see supplementary note, Supplementary Material online), and observed a simple estimator built on these estimated probabilities to be highly correlated with underlying simulated recombination rates (r=0.255, SE=0.002). We also note that the majority of our evaluations were based on data simulated by sampling from the coalescent with recombination, assuming neutral evolution. CoalNN was trained using distinct samples from the same process, while other methods rely on models devised to closely approximate the coalescent with recombination. In addition, these methods were often run using the same evolutionary parameters used to generate the data. Our estimates of performance are therefore obtained in idealized conditions and likely provide an optimistic picture of the performance of these methods compared to real data analyses. Addressing misspecification between simulated and real scenarios is an interesting direction of future work [also see Mo and Siepel (2023)]. We further note that our exploration of additional models, such as the Beta-coalescent, was limited to a small range of parameters. An additional limitation of this work is linked to our choice of architecture, which poses limits to CoalNN’s receptive field and may affect its performance in inferring very recent TMRCAs that involve long shared haplotypes. This was highlighted using saliency maps, where we observed nonzero gradients on the edges of the receptive field for sites with recent coalescence. Although we experimented with neural network architectures without observing significant performance gains (e.g., attention-based models Vaswani et al. 2017), several improvements are likely possible. In addition, our real data analyses used a CoalNN model trained on simulations that do not include NCGC events; experimenting with models that include NCGC is an interesting avenue of future work. Lastly, our approach for dating variants requires pairwise haplotype comparisons. This approach, and other methods we considered (Speidel et al. 2019; Albers and McVean 2020), scale quadratically with sample size. In addition to using CoalNN as a building block for other algorithms that efficiently infer full genealogies, as previously discussed, the development of extensions that jointly infer TMRCAs across several samples is an interesting direction of future work. Despite these limitations and areas of future development, we believe that the CoalNN model provides a valuable tool for the inference of coalescence times and allele ages and demonstrates the effectiveness of using simulation-trained models to analyze properties of gene genealogies.

## Supplementary Material

msad211_Supplementary_Data

## Data Availability

The CoalNN software, pretrained neural network weights, and allele age estimates are available at https://palamaralab.github.io/software/coalnn. Additional software, annotations, and data sets used in this study include: demographic models and genetic maps https://github.com/popgenmethods/pyrho; ASMC software https://github.com/PalamaraLab/ASMC; Relate software https://myersgroup.github.io/relate/; tsinfer software https://tsinfer.readthedocs.io/; tsdate software https://tsdate.readthedocs.io; GEVA software https://github.com/pkalbers/geva; Relate allele ages https://zenodo.org/record/3234689.; GEVA allele ages https://human.genome.dating; ${\mathrm{AMSC}}_{\mathrm{avg}}$ annotation https://palamaralab.github.io/software/asmc/data/; 1kGP Phase 3 data set (release 2013050) http://ftp.1000genomes.ebi.ac.uk/vol1/ftp/data_collections/1000G_2504_high_coverage/working/phase3_liftover_nygc_dir/; pathogenicity annotations http://ftp.1000genomes.ebi.ac.uk//vol1/ftp/release/20130502/supporting/functional_annotation/filtered/; LDSC baseline model https://alkesgroup.broadinstitute.org/LDSCORE/; LDSC software https://github.com/bulik/ldsc. Data analyses are based on open-source libraries and software programs that are available online: Scipy (Virtanen et al. 2020), Matplotlib (Hunter 2007), NumPy (Oliphant 2006; Van Der Walt et al. 2011), Pandas (McKinney 2010; The pandas development team 2020), and PyTorch (Paszke et al. 2019).
