# Supplementary material for: Inference of Coalescence Times and Variant Ages Using Convolutional Neural Networks
Source: Mol Biol Evol. 2023 Sep 20;40(10):msad211. doi: 10.1093/molbev/msad211 (PMC10581698; doi:10.1093/molbev/msad211)
Supplement: msad211_Supplementary_Data [file msad211_supplementary_data.pdf]

Supplementary Information
Inference of coalescence times and variant ages using convolutional neural networks

Juba Nait Saada, Zoi Tsangalidou, Miriam Stricker, Pier Francesco Palamara

Supplementary Note

Additional details on CoalNN's network architecture

We apply a convolutional neural network on genomic windows of fixed length L, where L is the number of sites necessary to cover the first 10 centimorgans (cM) of Chromosome 2, since this provides a receptive field large enough to capture long identical-by-descent segments as explained in the intepretability section. We selected the beginning of Chromosome 2 as a representative region due to its recombination rate being close to the genome-wide average (1.66 cM per Mb in the first 30 Mb; mean = 1.45 cM per Mb, s.d. = 0.33 across the autosomes (Palamara et al., 2018)). We measure genomic regions using cM, rather than base pairs, to account for uneven recombination rates along the genome, as recombination is the primary determinant of changes in TMRCA. During training, recombination maps from different chromosomes are sampled at random to avoid overfitting to chromosome 2. Rather than using padding, we add a fixed amount of genomic data as context on either side of the input sequence, resulting in a region of length $L_1 > L$.

The genotype data provided in input to CoalNN is encoded as x_i, $i \in \{1, ..., L_1\}$, where x_i takes floating point values. For simulated SNP array and sequencing data $x_i \in \{0, 1\}$ indicates whether the haploid individual carries an ancestral or a derived allele, while in experiments involving imputed data, x_i takes continuous dosage values in $[0, 1]$, reflecting imputation uncertainty. For binary data, we compute the AND and the XOR functions between the two haplotypes, which reflect the presence of mismatching or shared alleles, respectively. We also performed experiments in which we trained a model by providing the raw sequencing data as input directly, without applying the AND and XOR functions. For imputation experiments, we trialled two approaches: using the model trained on sequencing data after rounding the continuous dosage to binary values, or training a new model on dosages from simulated imputed data, allowing the network to take floating-point values in input.

We first apply a batch normalization layer and stack five convolution blocks (ConvBlocks), each consisting of a convolution layer, a batch normalization layer, and a ReLU activation function. The first convolution block aims to capture long-range dependencies by using a large kernel size while subsequent layers focus on smaller and smaller windows (kernel sizes of 701, 201, 51, 7, 3 respectively). These kernel sizes were chosen to allow the first layers of the network to have large receptive fields, without significantly increasing the number of trainable parameters. Since small TMRCAs correspond to shared ancestry in the recent past, they are characterized by long (e.g., > 1 cM) and nearly identical haplotype segments. To capture these segments, we increased the receptive field of the network by utilizing a dilated convolution in the first block, which effectively enlarges the convolution kernel by inserting gaps between kernel elements (Yu and Koltun, 2015). The dilation factor is automatically tuned so that the receptive field approximately corresponds to the first 5 cM of Chromosome 2. A convolutional layer goes through all input channels in the previous layer and the number of channels in each layer is incrementally increased to allow for increasingly complex feature extraction. At the same time, since a convolution operation can be seen as summarizing nearby elements in the input, after each convolution block, the sequence length is reduced until ultimately reaching L ($L_1 > L_2 > L_3 > L_4 > L_5 > L$). We finally apply a 1×1 convolution layer to reduce the channel dimension. The resulting output is a $L \times 1 \times 2$ tensor, containing the TMRCA estimates and the unscaled estimated probabilities (logits) of observing a recombination breakpoint for each of the L sites, i.e., for every genomic site the network predicts the TMRCA and whether that site is a recombination breakpoint, resulting in $2 \times L$ predictions. When deploying CoalNN for inference, we apply an additional scaling layer on both estimates: predicted TMRCAs that exceed the maximum coalescence time observed while training are clipped and a softmax function is applied to the logits to turn them into estimated probabilities of recombination. Clipping is not applied to the predictions of ASMC, since the HMM discretizes time, producing a bounded output.

Additional simulation experiments

We performed experiments where we assumed recombination rates to be unknown a priori. We trained CoalNN using $N_e = 10K$ and a constant recombination rate of $\rho = 1$cM/Mb, while other evolutionary parameters were set as described in the Methods section. We then simulated distinct data sets using recombination rates from Chromosome 2, and aimed to estimate the unknown underlying simulated recombination rate between each pair of neighboring sites, $\hat{\rho}_i$. At every position i along the genome and for every pair of haplotypes j, CoalNN outputs an estimate of TMRCA $\hat{t}_{i,j}$ and the estimated probability of recombination $\hat{p}_{i,j}$. We estimated recombination rates using $\hat{\rho}_i = N^{-1} \sum_{j=0}^{N} \hat{p}_{i,j} / \hat{t}_{i,j}$, which accounts for the fact that the probability of observing a recombination event depends approximately linearly on $\rho_i t_{i,j}$, where ρ_i is the true recombination rate at position i and $t_{i,j}$ is the true TMRCA for the pair. We observed these estimates to be highly correlated with the true underlying genetic distance between consecutive polymorphisms ($r = 0.255$, SE$= 0.002$ across 5 distinct random seeds).

Supplementary Figures

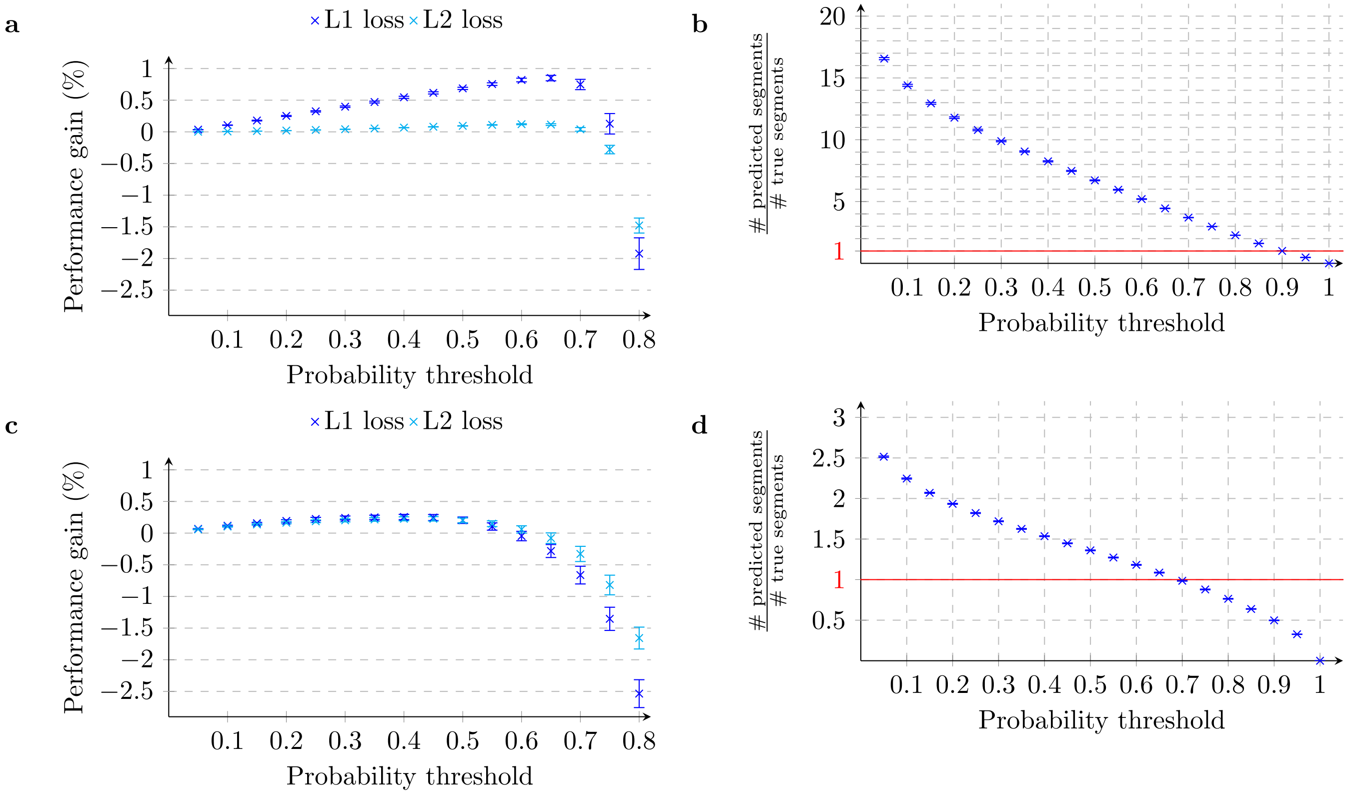

Supplementary Fig. 1. Forming piecewise constant TMRCA estimates. a, c. In **b** (resp in **d**) we report the average percent improvement of piecewise constant TMRCA prediction over the raw output of CoalNN using L1 (mean absolute error: MAE) and L2 (root mean squared error: RMSE) losses for sequencing (resp. array) data, for increasing threshold values. **b, d.** In **c** (resp. in **e**) we report the average ratio between the total number of predicted segments and the total number of true segments for increasing threshold values, where a segment refers to a piece of the genome with constant TMRCA value. Error bars represent standard errors across 10 simulations.

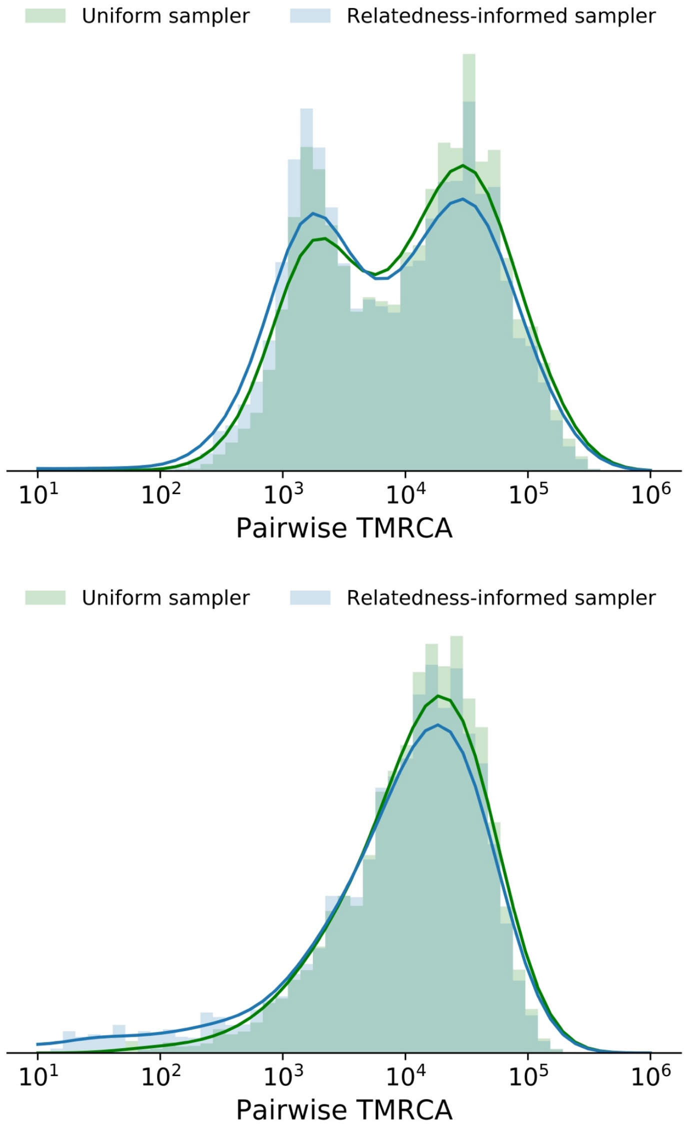

Supplementary Fig. 2. Pairwise TMRCA sampling distribution. Distributions of pairwise TMRCA in generations (empirical histograms) obtained from a uniform sampler (in green) and a relatedness-informed sampler (in blue) from a single simulation under a European demographic model (top) and a constant population size $N_e = 10,000$ (below). Solid curves show kernel density estimations using Gaussian kernels (bandwidth $= 0.25$).

a

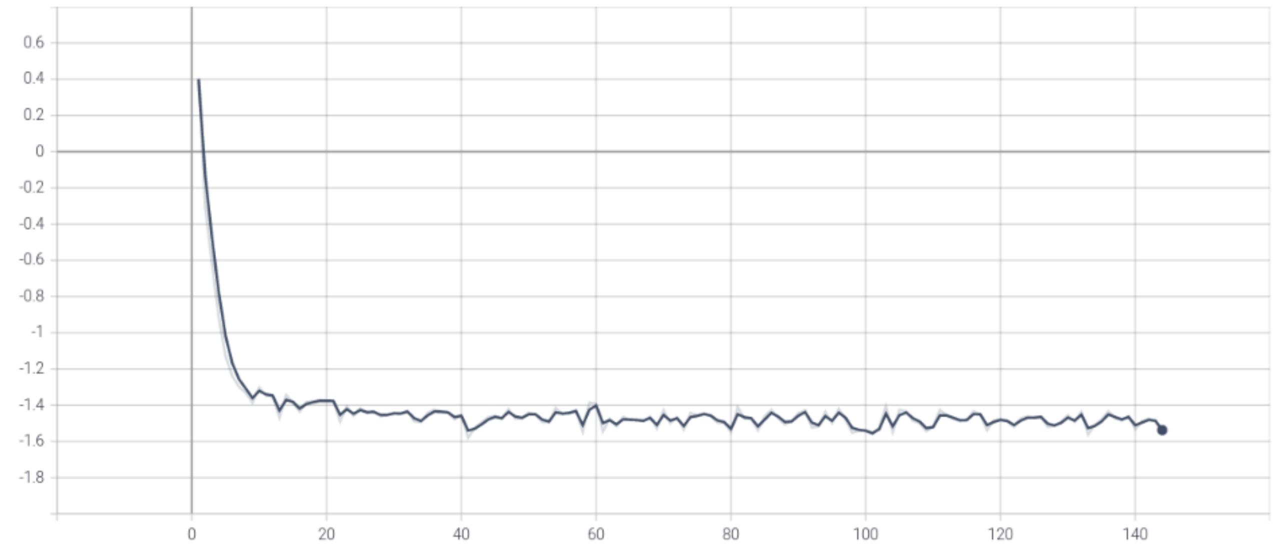

b

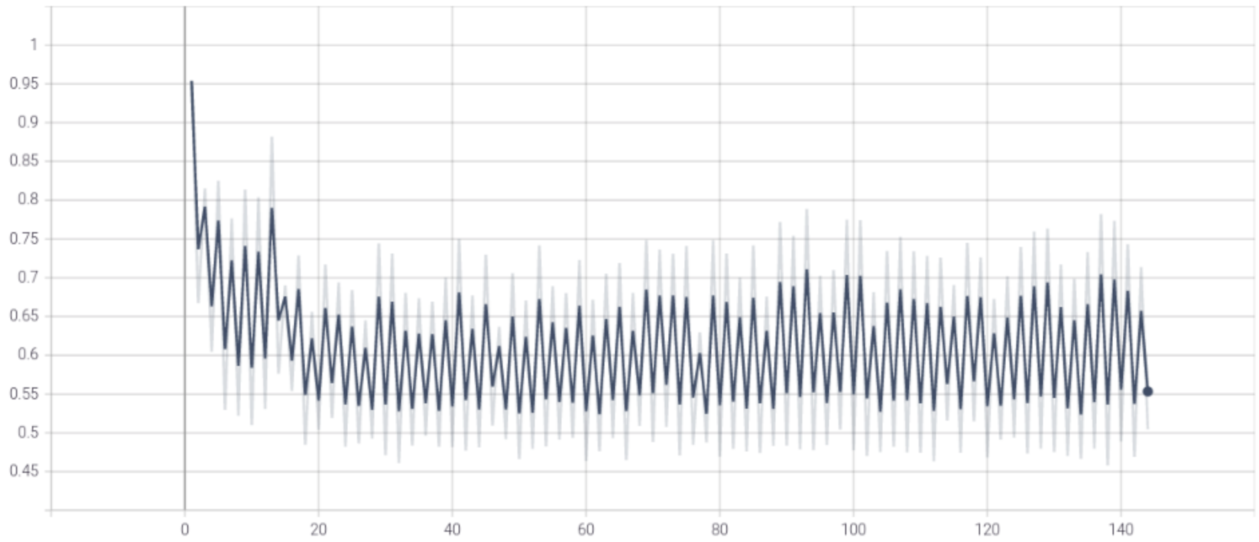

Supplementary Fig. 3. CoalNN training and validation loss. Training (**a**) and validation (**b**) loss obtained during the training of CoalNN on a constant population size demography with $N_e = 10{,}000$. Y axes represent loss values, X axes represent number of training epochs.

a

b

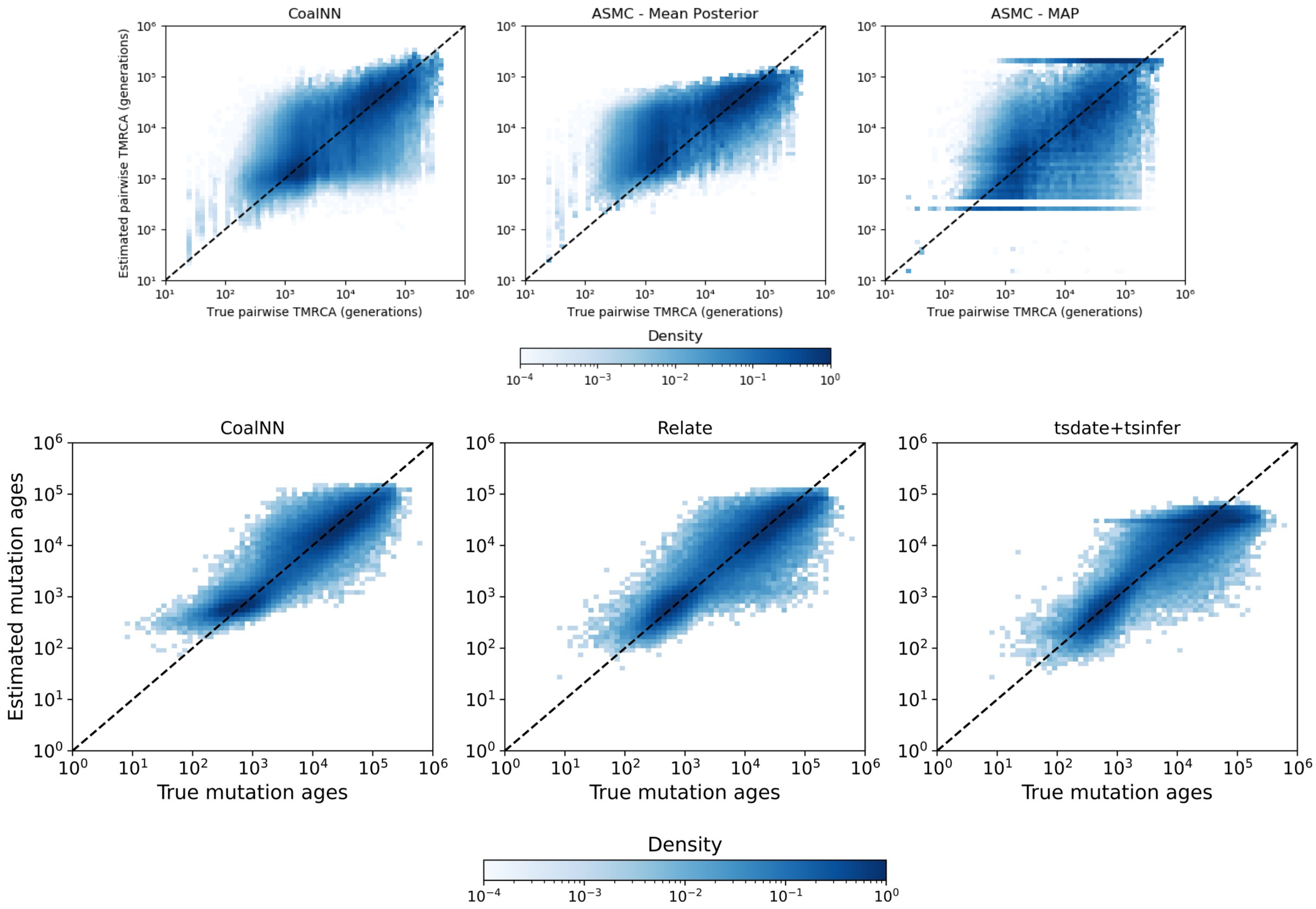

Supplementary Fig. 4. Pairwise TMRCA and allele age prediction under a European demographic model. a. True pairwise TMRCAs (x axis) versus those estimated by CoalNN and ASMC (y axis) on array data for one simulation. **b.** True non-singleton variant ages (x axis) versus those estimated by CoalNN, Relate and tsinfer+tsdate (y axis) on sequencing data for one simulation.

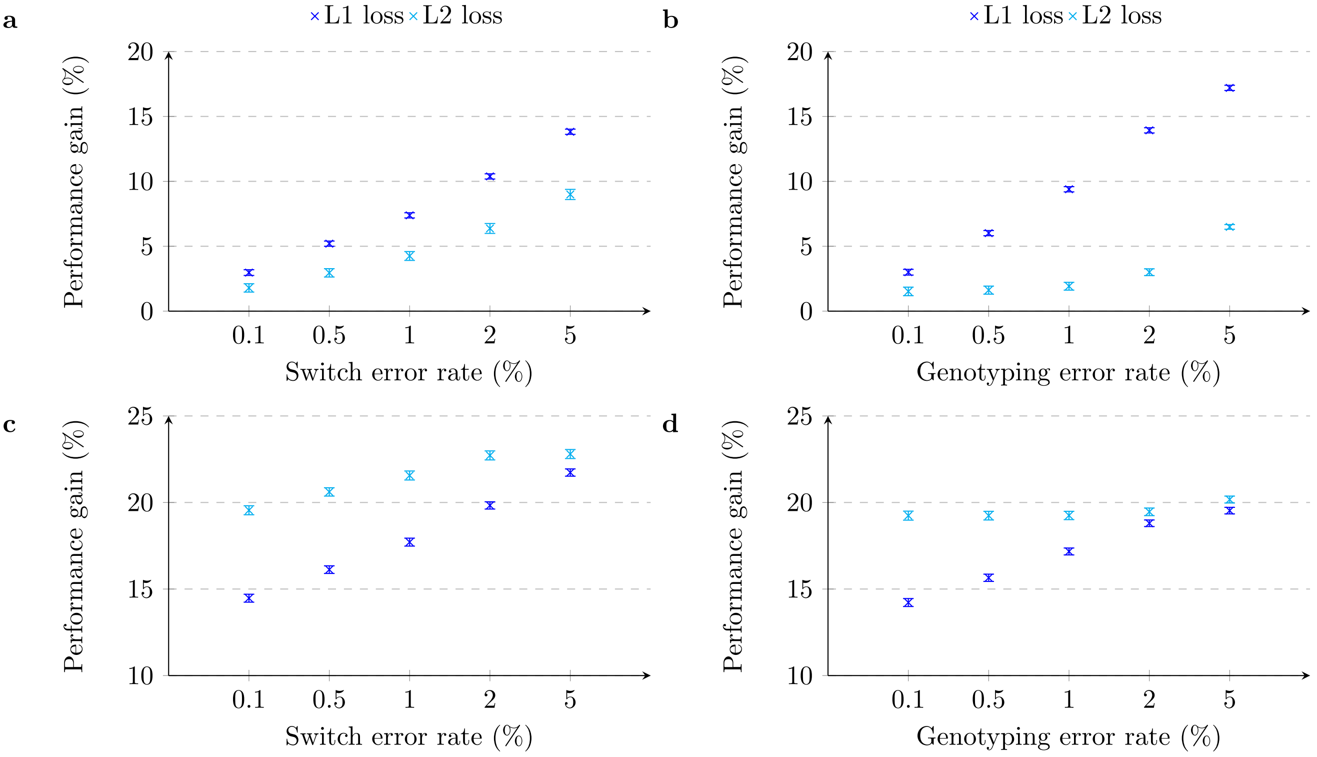

Supplementary Fig. 5. Robustness to genotyping and phasing errors. Average percent performance improvement for TMRCA prediction in sequencing data of CoalNN over ASMC mean posterior in **a, b** and over ASMC MAP in **c, d** for the L1 (mean absolute error: MAE) and L2 (root mean squared error: RMSE) losses for increasing switch and genotyping error rates respectively. Error bars represent standard errors across 20 simulations.

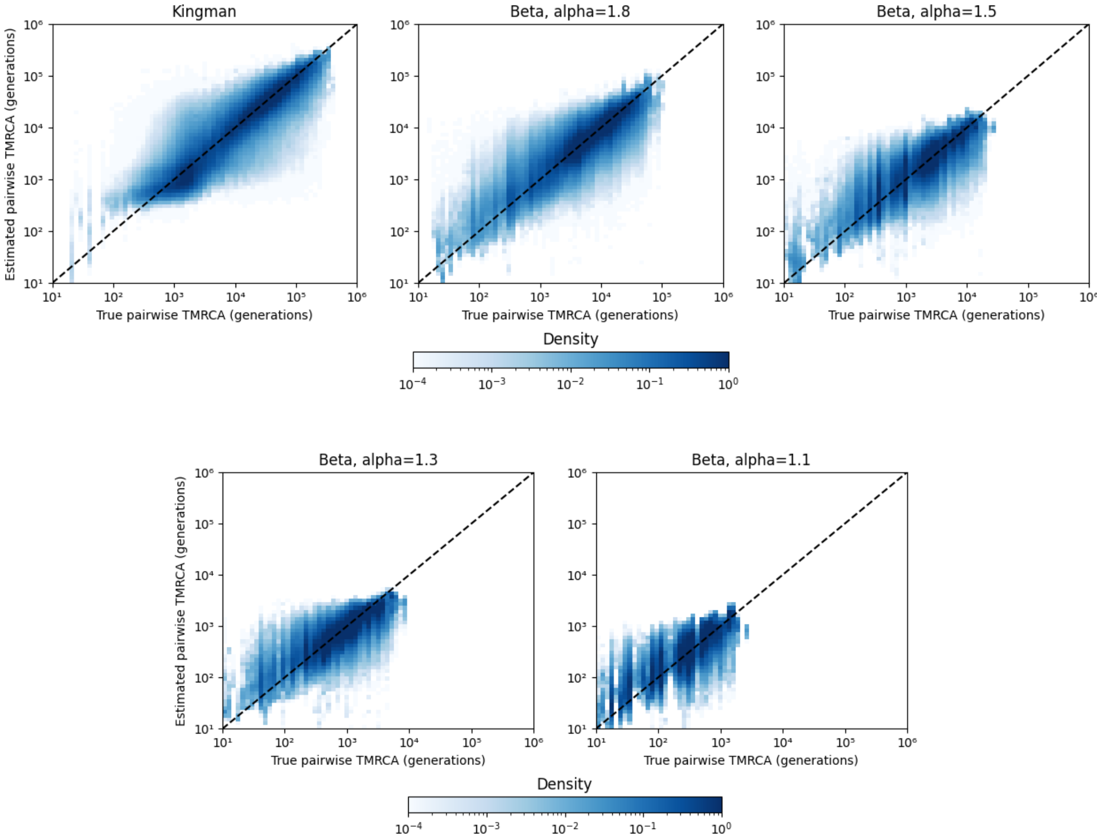

Supplementary Fig. 6. Scatter plots of true vs CoalNN-predicted TMRCA. The evolutionary model and parameters used are: Kingman coalescent with $N_e = 10{,}000$ (top left), Beta coalescent with alpha $= 1.8$ and $N_e = 20{,}000$ (top middle), Beta coalescent with $N_e = 50{,}000$ and alpha $= 1.5$ (top right), alpha $= 1.3$ (bottom left) and alpha $= 1.1$ (bottom right).

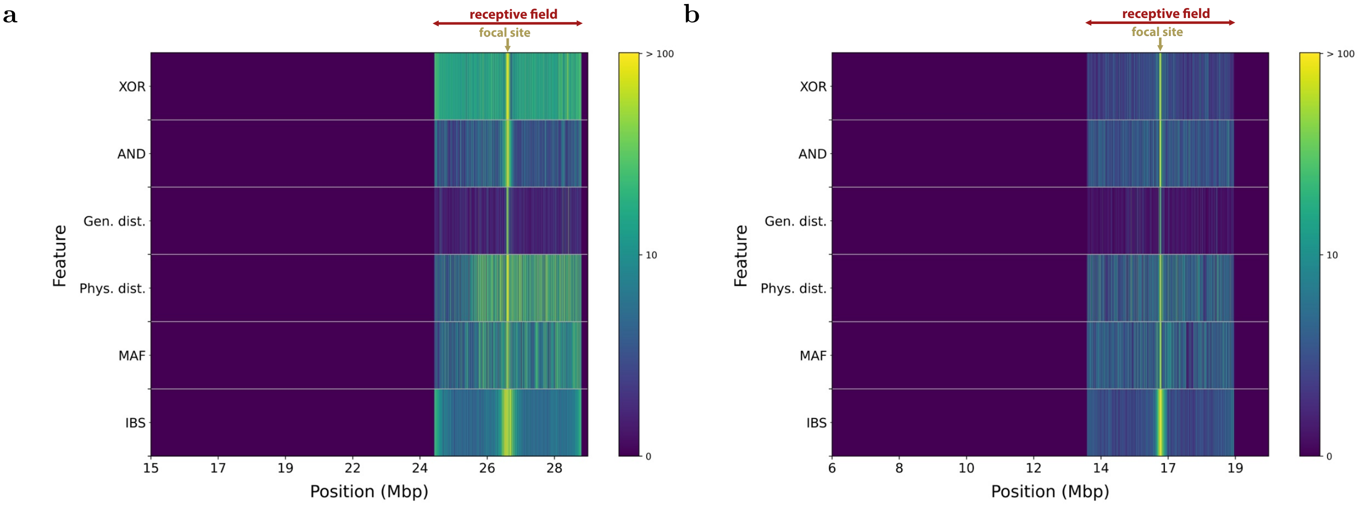

Supplementary Fig. 7. Feature importance analysis. We obtained saliency maps (Simonyan et al., 2014; Zeiler and Fergus, 2014) at a genomic locus with recent coalescence time in **a** (TMRCA = 37 generations) and with deep coalescence time in **b** (TMRCA = 237,079 generations). Values are shown in log scale and correspond to $|\text{input} \times \text{gradient}|$ (Shrikumar et al., 2016), where input refers to the batch normalized input (see Methods). Higher values (closer to yellow) indicate input positions with large effects on the TMRCA estimate. Positions with value 0 have no impact on the prediction and are typically outside of the receptive field.

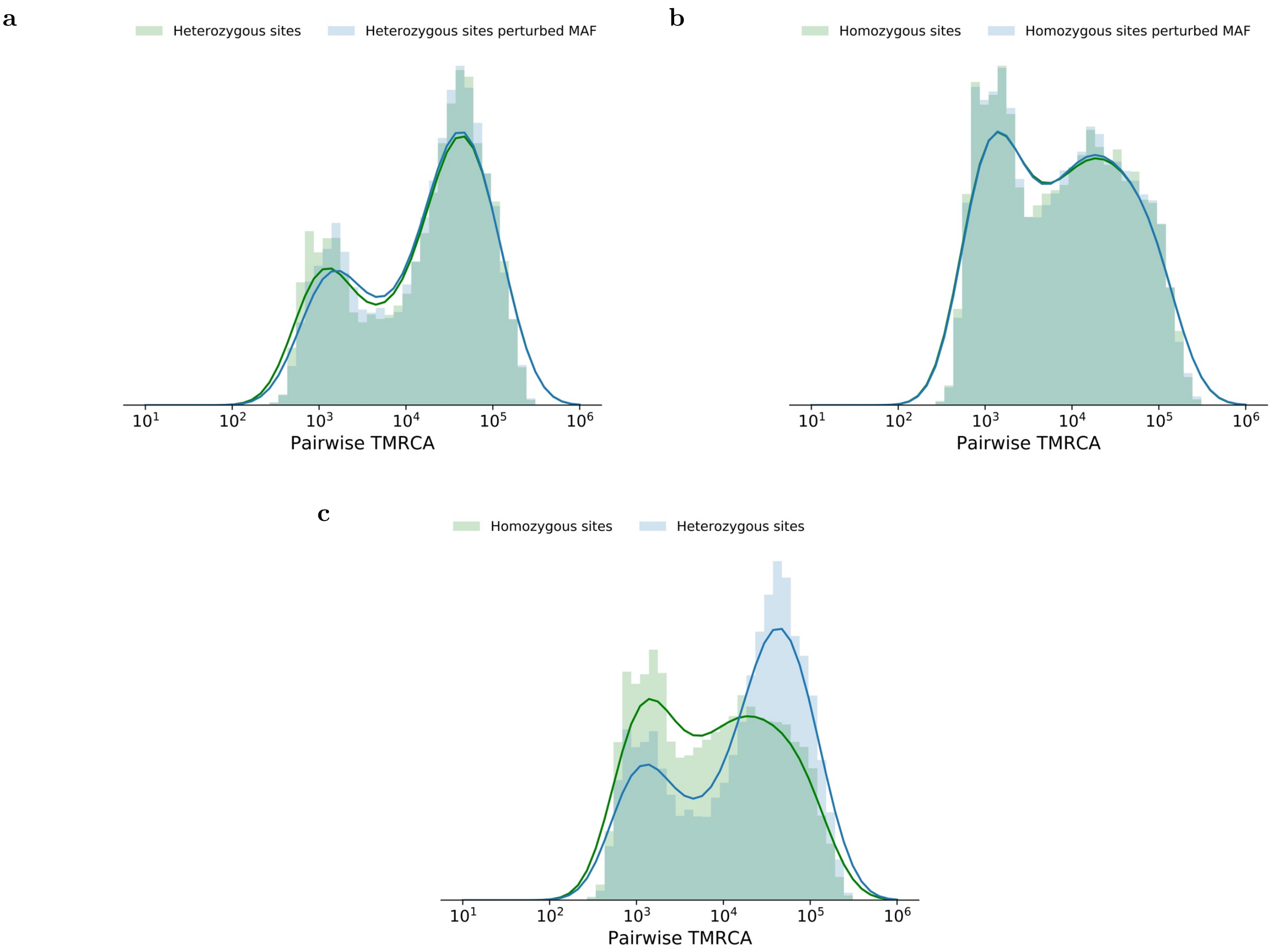

Supplementary Fig. 8. Predicted TMRCA distributions under MAF perturbation. Empirical histograms showing the predicted TMRCAs at heterozygous (in **a**) or at homozygous derived (in **b**) genomic sites before and after the MAF perturbation, for one simulation. Increasing the input MAF value by 5% at sites for which individuals are heterozygous resulted in an average increase in predicted TMRCA of 181.5 (SE=3.7) generations. Decreasing the MAF at homozygous sites resulted in an average decrease of predicted TMRCA of 27.6 (SE=1.7) generations. **c** shows TMRCA values predicted by CoalNN at heterozygous and homozygous derived sites, without any MAF perturbation. Solid curves show kernel density estimations using Gaussian kernels (bandwidth = 0.25).

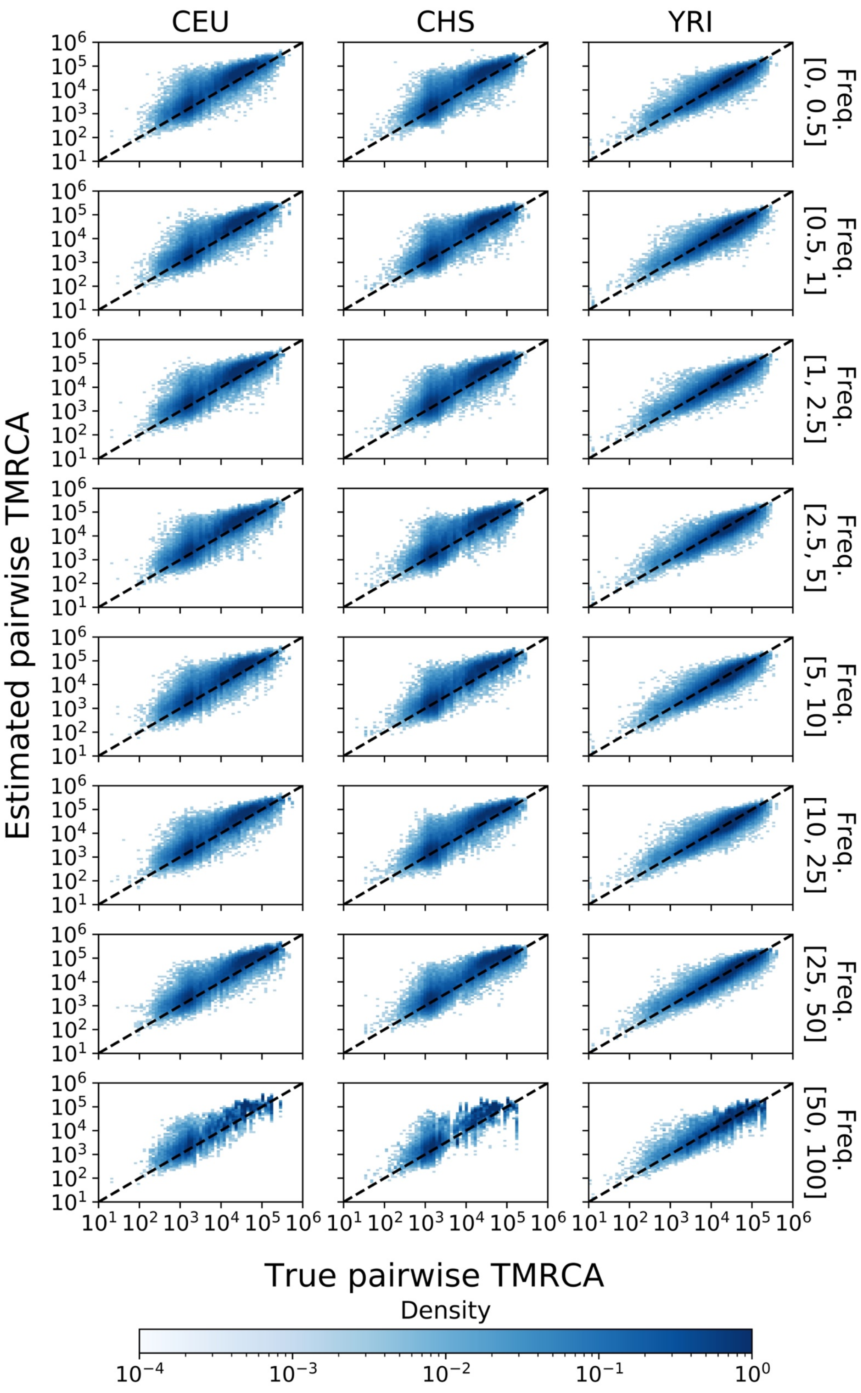

Supplementary Fig. 9. Performance of CoalNN trained on a constant demographic prior. We visualise true pairwise TMRCAs (x axis) versus those estimated by CoalNN trained on a constant demographic population size (N_e = 20,000, y axis), stratified by derived allele frequency, on simulations using recombination rates and demographic history from CEU, CHS and YRI (Terhorst et al., 2017; Spence and Song, 2019) (sequencing data for 150 diploid individuals and a 30 Mbp region from chromosome 2).

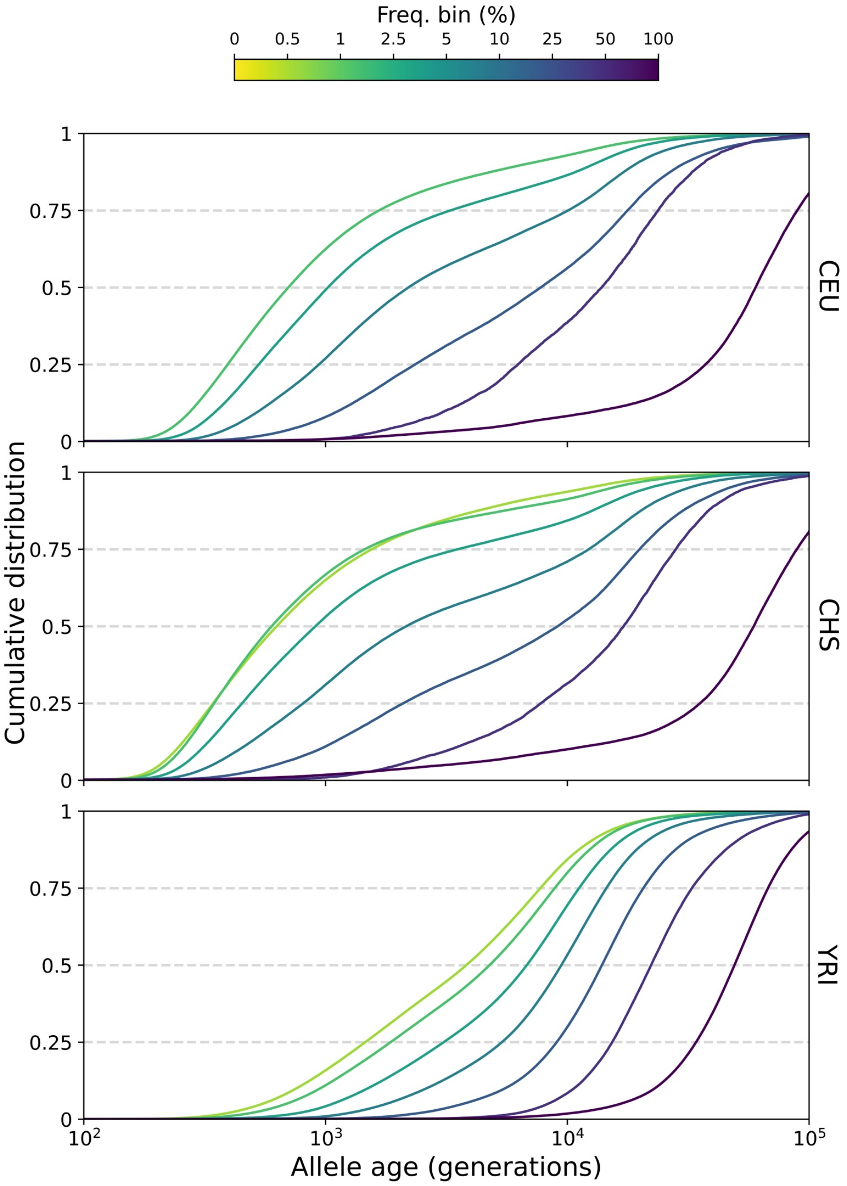

Supplementary Fig. 10. Age distribution of variants dated by CoalNN trained on a constant demographic prior. Cumulative age distribution of genome-wide variants dated by CoalNN trained on a constant demographic population size ($N_e = 20{,}000$), stratified by derived allele frequency as observed within a given population group. See Supplementary Table 9 for additional information.

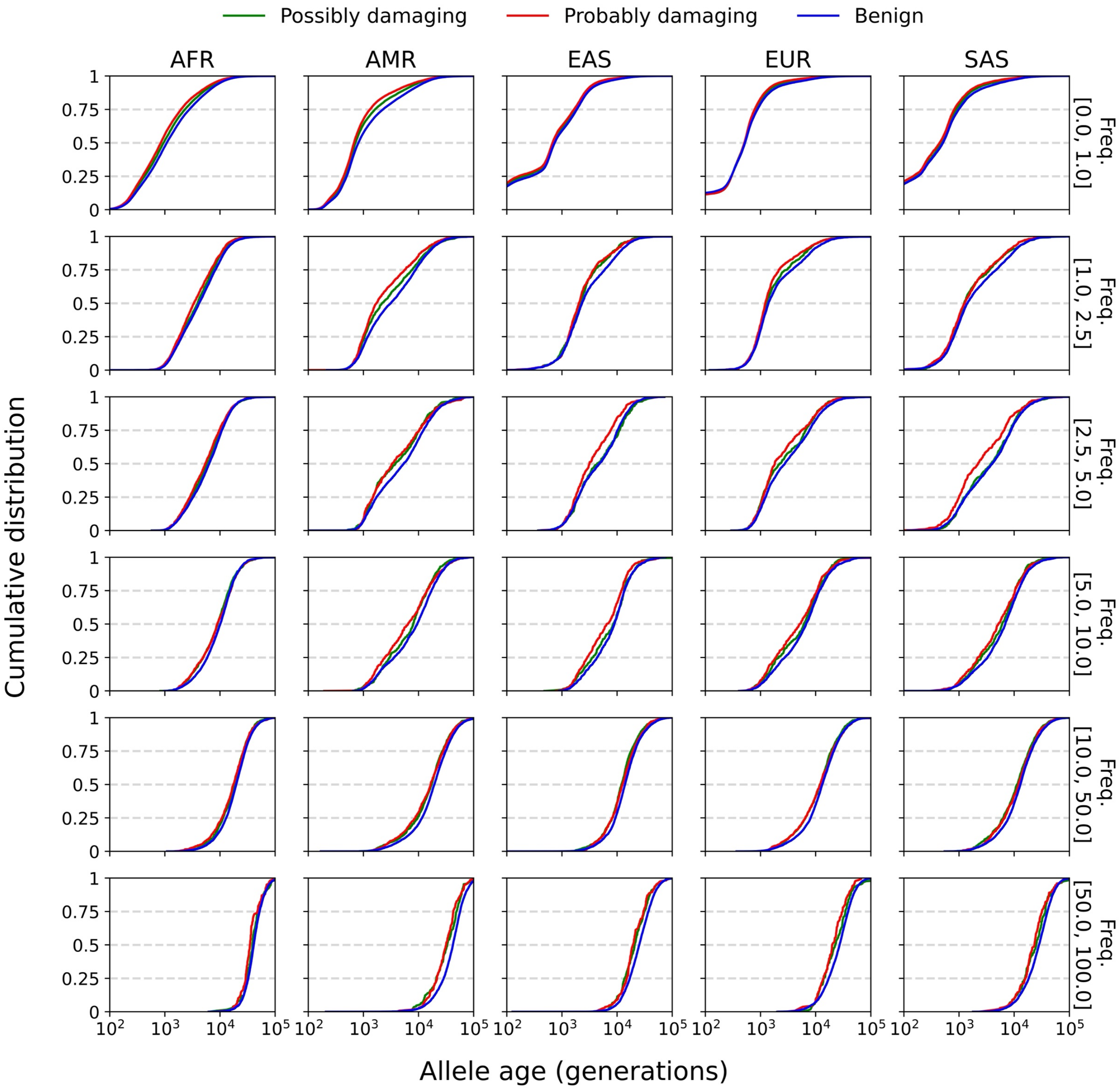

Supplementary Fig. 11. Allele age distribution of variants annotated by PolyPhen-2. Cumulative distribution function of allele ages inferred by CoalNN for different variant pathogenicity levels predicted using PolyPhen-2 ("possibly damaging", "probably damaging" or "benign") (Adzhubei et al., 2010), stratified by derived allele frequency within each population group.

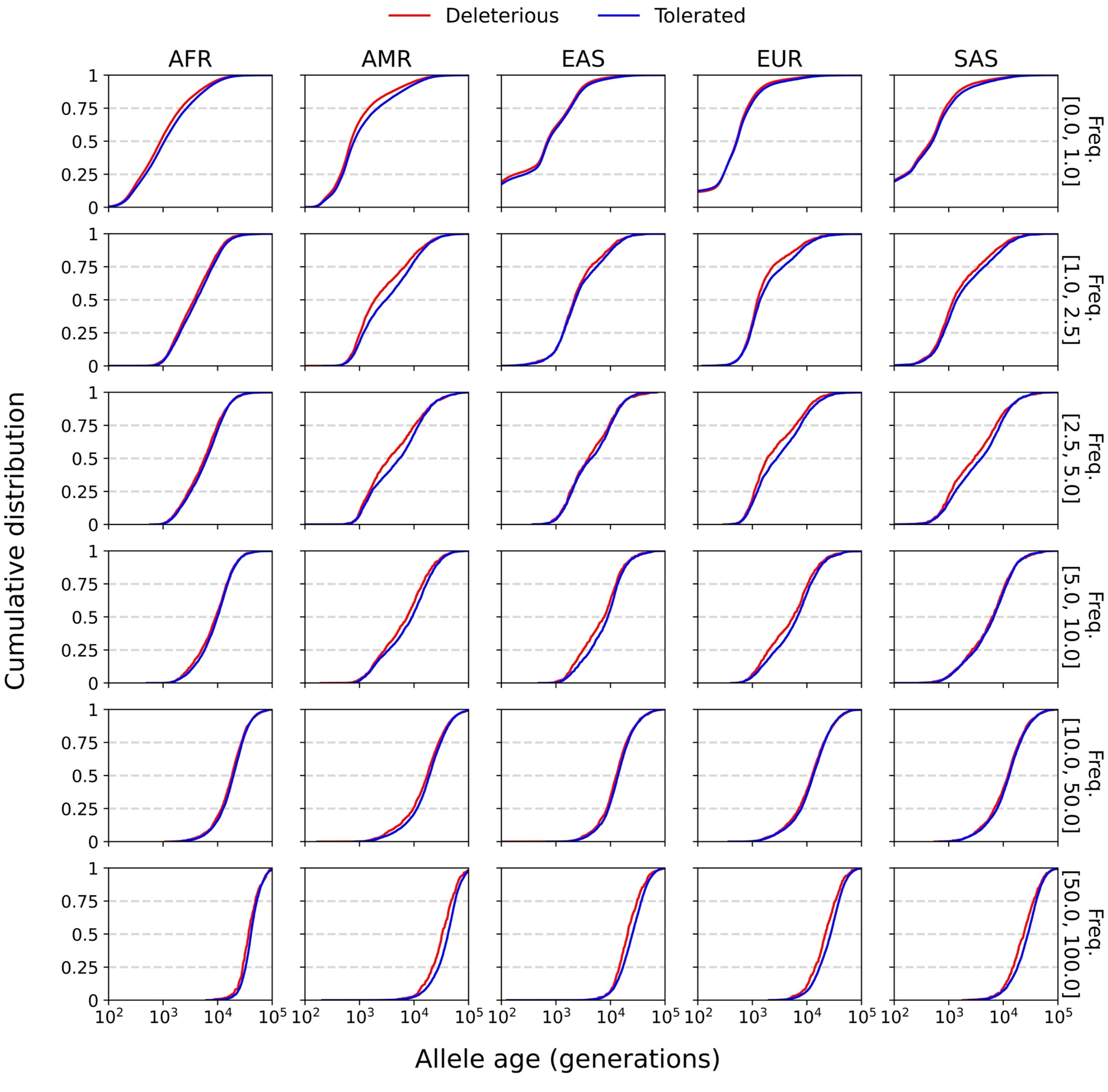

Supplementary Fig. 12. Allele age distribution of variants annotated by SIFT. Cumulative distribution function of allele ages inferred by CoalNN for different variant pathogenicity levels predicted using SIFT ("deleterious" or "tolerated") (Sim et al., 2012), stratified by derived allele frequency within each population group.

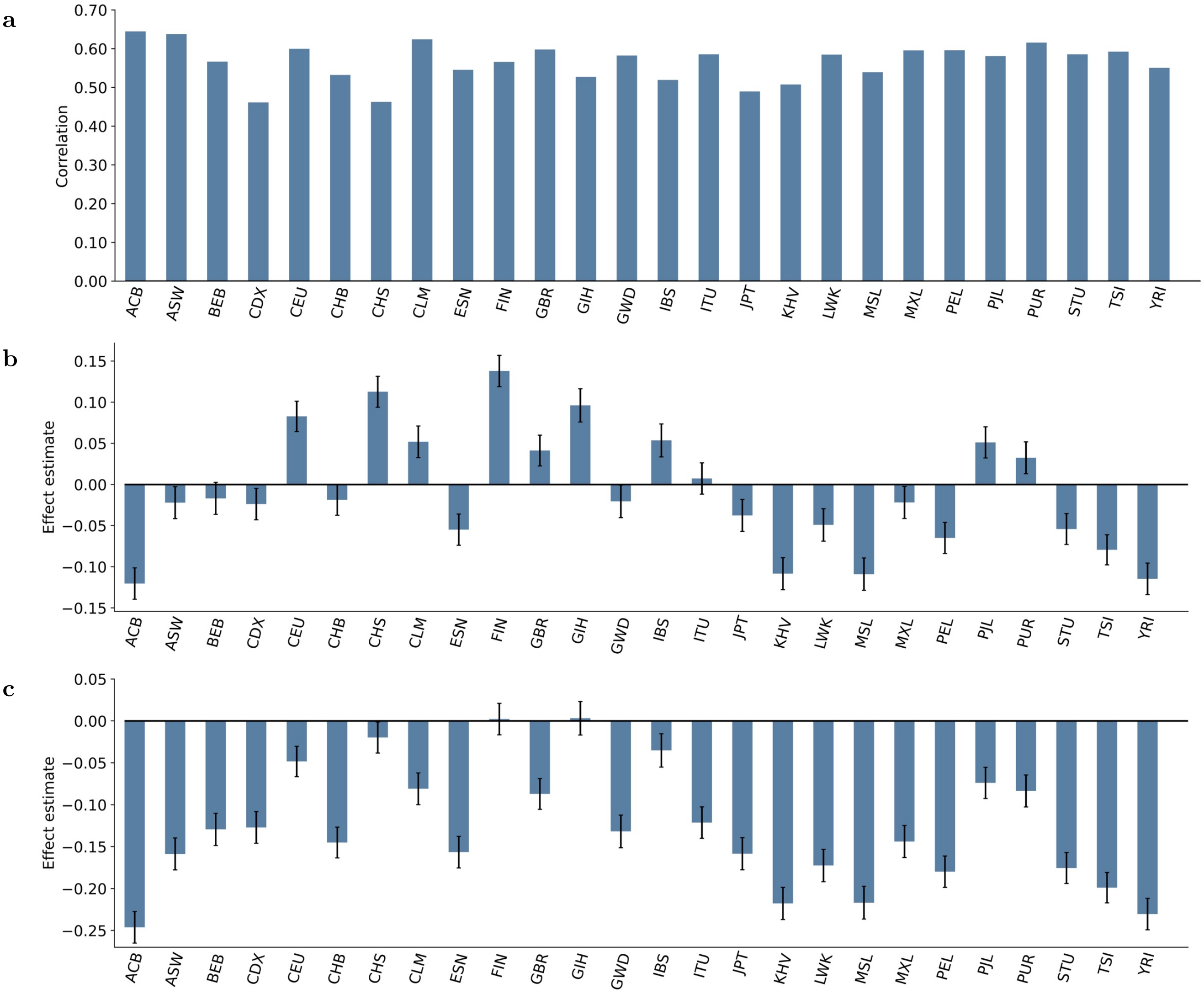

Supplementary Fig. 13. S-LDSC analyses including both ARGweaver and CoalNN. a. Correlations between each of the 26 population-specific MAF-adjusted CoalNN annotations and the MAF-adjusted ARGweaver annotation. **b, c.** τ^* estimates (meta-analysed across 63 independent diseases and complex traits) of CoalNN MAF-adjusted allele age annotation on each of the 26 populations in marginal independent S-LDSC analyses conditioned on the full 97 Baseline-LD model in **b** and conditioned on 96 Baseline-LD annotations (all but ARGweaver) in **c**.

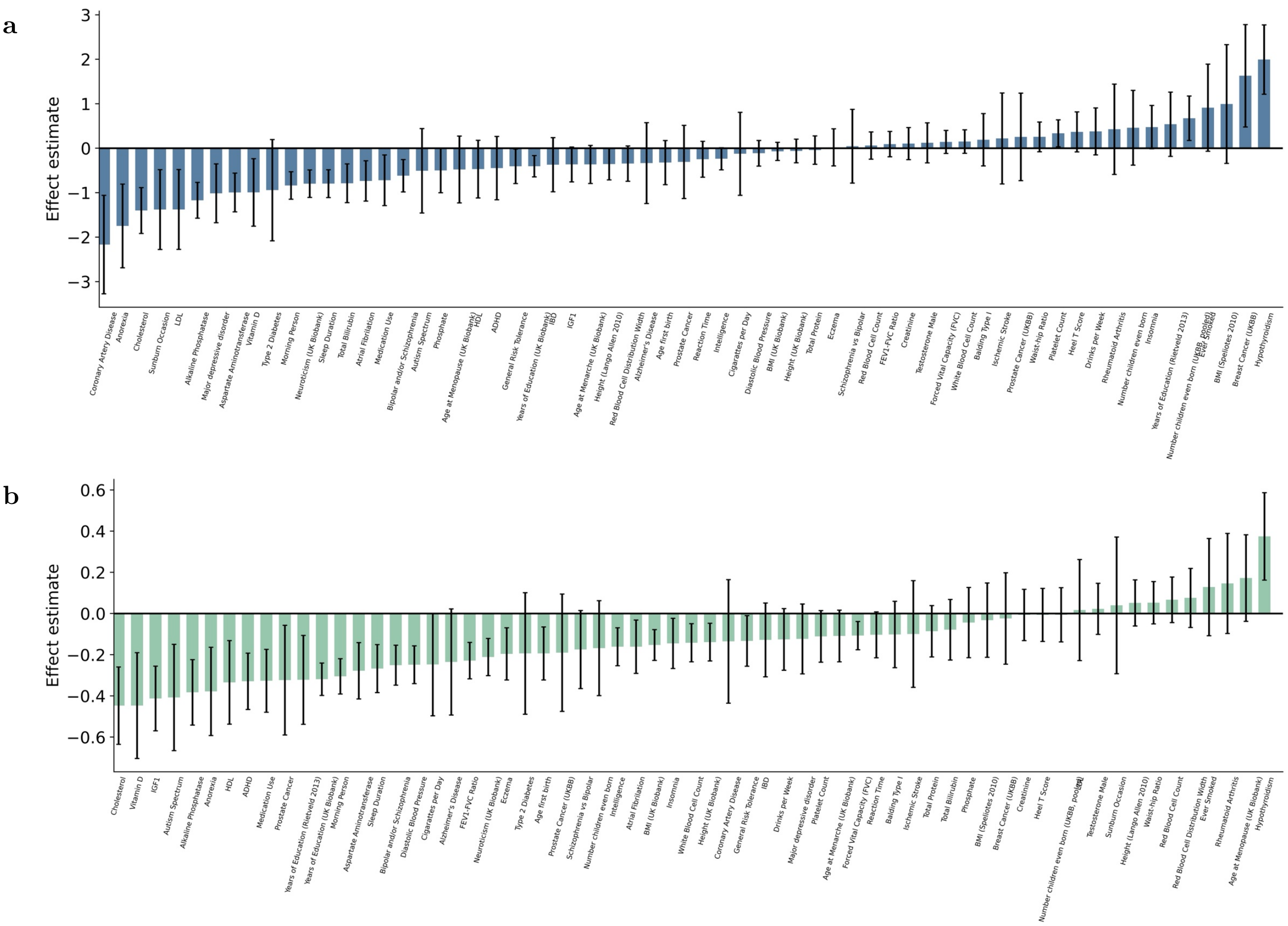

Supplementary Fig. 14. CoalNN heritability effect for individual traits. S-LDSC τ^* value of the CoalNN MAF-adjusted annotation (averaged across 26 populations) in **a** and of the ARGweaver MAF-adjusted annotation in **b** for 63 independent diseases and traits (listed in Supplementary Table 2), conditioned on 96 baselineLD annotations. Error bars represent s.e. of the τ^* estimate.

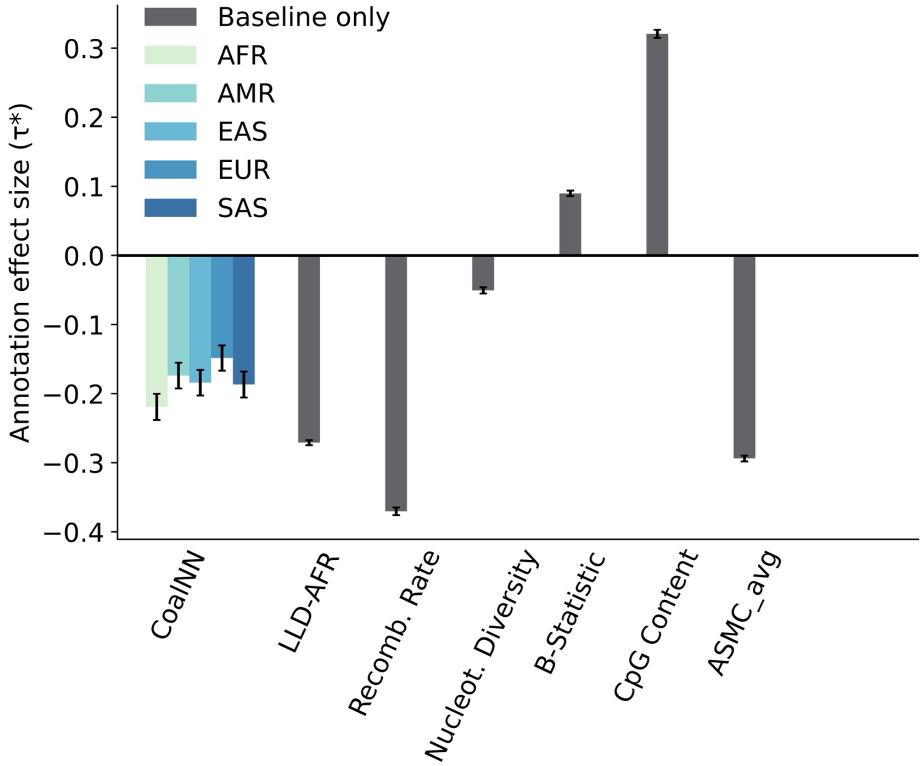

Supplementary Fig. 15. S-LDSC analysis of CoalNN annotations built using population groups. S-LDSC τ^* estimates of CoalNN annotations built using each population group (AFR, AMR, EAS, EUR and SAS) in independent S-LDSC analyses conditioned on 96 baselineLD annotations (all but ARGweaver), meta-analyzed across 63 independent disease and traits (listed in Supplementary Table 2). For comparison, we also report τ^* effects of other Baseline-LD evolutionary annotations (level of LD measured in African populations, recombination rate, nucleotide diversity, McVicker B-statistic and average pairwise TMRCA ASMC_{avg}) before the introduction of the CoalNN annotations (in dark grey). Adding the CoalNN annotations did not result in a significant change of τ^* for other annotations. Error bars represent standard errors of the meta-analyzed τ^* estimates. Numerical results are reported in Supplementary Table 12.

Supplementary Tables

Population Code	Description	Group Code
CHB	Han Chinese in Beijing, China	EAS
JPT	Japanese in Tokyo, Japan	EAS
CHS	Han Chinese South	EAS
CDX	Chinese Dai in Xishuangbanna, China	EAS
KHV	Kinh in Ho Chi Minh City, Vietnam	EAS
CEU	Utah residents (CEPH) with Northern and Western European ancestry	EUR
TSI	Toscani in Italy	EUR
FIN	Finnish in Finland	EUR
GBR	British in England and Scotland	EUR
IBS	Iberian populations in Spain	EUR
YRI	Yoruba in Ibadan, Nigeria	AFR
LWK	Luhya in Webuye, Kenya	AFR
MAG	Gambian in Western Division, The Gambia - Mandinka	AFR
MSL	Mende in Sierra Leone	AFR
ESN	Esan in Nigeria	AFR
ASW	African Ancestry in Southwest US	AFR
ACB	African Caribbean in Barbados	AFR
MXL	Mexican Ancestry in Los Angeles, California, US	AMR
PUR	Puerto Rican in Puerto Rico	AMR
CLM	Colombian in Medellin, Colombia	AMR
PEL	Peruvian in Lima, Peru	AMR
GIH	Gujarati Indians in Houston, Texas, US	SAS
PJL	Punjabi in Lahore, Pakistan	SAS
BEB	Bengali in Bangladesh	SAS
STU	Sri Lankan Tamil in the UK	SAS
ITU	Indian Telugu in the UK	SAS

Supplementary Table 1. 1kG populations. Population labels and supergroup assignments. (Retrieved from www.internationalgenome.org (Byrska-Bishop et al., 2022))

Phenotype	Reference	h_g^2	Z-score	N
BMI (UK Biobank)	UK Biobank	0.278	41.478	457,824
Years of Education (UK Biobank)	UK Biobank	0.136	35.789	454,813
Forced Vital Capacity (FVC)	UK Biobank	0.234	32.444	371,949
Intelligence	Savage et al. 2018 Nat Genet	0.186	29.047	264,193
Neuroticism (UK Biobank)	UK Biobank	0.114	28.475	372,066
Diastolic Blood Pressure	UK Biobank	0.233	27.738	422,771
White Blood Cell Count	UK Biobank	0.227	27.386	444,502
Height (UK Biobank)	UK Biobank	0.674	25.824	458,303
Morning Person	UK Biobank	0.106	24.674	410,520
Age at Menarche (UK Biobank)	UK Biobank	0.253	24.563	242,278
TotalProtein	UK Biobank	0.181	24.147	397,652
Waist-hip Ratio	UK Biobank	0.171	23.681	458,417
FEV1-FVC Ratio	UK Biobank	0.276	23.615	371,949
Reaction Time	Davies et al. 2018 Nat Comm	0.081	22.556	300,486
Creatinine	UK Biobank	0.221	21.911	434,158
Red Blood Cell Count	UK Biobank	0.261	21.775	445,174
Bipolar and/or Schizophrenia	Ruderfer et al. 2018 Cell	0.334	21.656	107,620
Platelet Count	UK Biobank	0.351	21.242	444,382
Heel T Score	UK Biobank	0.355	20.514	445,921
Sleep Duration	Dashti et al. 2019 Nat Comm	0.071	19.667	446,118
IGF1	UK Biobank	0.288	18.682	432,292
General Risk Tolerance	Karlsson Linner et al. 2019 Nat Genet	0.051	18.357	466,571
AspartateAminotransferase	UK Biobank	0.114	18.306	430,982
Red Blood Cell Distribution Width	UK Biobank	0.217	17.803	442,700
Insomnia	Jansen et al. 2019 Nat Genet	0.044	17.600	383,948
Height (Lango Allen 2010)	Lango Allen et al. 2010 Nature	0.222	17.512	131,547
Number children even born (UKBB, pooled)	UK Biobank	0.039	16.783	456,500
Atrial Fibrilation	Nielsen et al. 2018 AJHG	0.022	16.769	1,030,836
TestosteroneMale	UK Biobank	0.184	16.577	196,813
BMI (Speliotes 2010)	Speliotes et al., 2010 Nat Genet	0.146	16.233	122,033
Hypothyroidism	UK Biobank	0.055	15.167	459,324
Age first birth	Barban et al., 2016 Nat Genet	0.062	15.073	222,037
Balding Type I	UK Biobank	0.223	14.697	208,336
AlkalinePhosphatase	UK Biobank	0.235	14.675	433,862
Eczema	UK Biobank	0.085	14.603	458,699
Drinks per Week	Liu et al. 2019 Nat Genet	0.046	14.438	527,299
Phosphate	UK Biobank	0.129	14.198	397,561
Medication Use	Wu et al. 2019 Nat Comm	0.157	14.036	78,808
IBD	de Lange et al. 2017 Nat Genet	0.321	13.193	59,957
VitaminD	UK Biobank	0.086	12.464	415,700
Major depressive disorder	Wray et al. 2018 Nat Genet	0.073	12.339	170,229
Cholesterol	UK Biobank	0.131	11.670	435,137
ADHD	Demontis et al. 2019 Nat Genet	0.248	11.634	55,374
TotalBilirubin	UK Biobank	0.084	11.066	429,423
Age at Menopause (UK Biobank)	UK Biobank	0.109	11.040	143,025
Sunburn Occasion	UK Biobank	0.073	9.618	344,229
Number children even born	Barban et al., 2016 Nat Genet	0.023	9.542	318,863
Schizophrenia vs Bipolar	Ruderfer et al. 2018 Cell	0.265	9.495	38,855
Years of Education (Rietveld 2013)	Rietveld et al., 2013 Science	0.077	9.354	126,559
HDL	Teslovich et al., 2010 Nature	0.125	9.259	97,749
LDL	Teslovich et al., 2010 Nature	0.103	8.821	93,354
Cigarattes per Day	Liu et al. 2019 Nat Genet	0.058	8.657	257,073
Ischemic Stroke	Malik et al. 2018 Nat Genet	0.017	8.350	440,328
Anorexia	Boraska et al., 2014 Mol Psych	0.236	8.274	32,143
Alzheimer's Disease	Jansen et al. 2019 Nat Genet	0.014	7.833	433,886
Rheumatoid Arthritis	Okada et al., 2014 Nature	0.187	7.510	37,681
Ever Smoked	TAG Consortium, 2010 Nat Genet	0.085	7.482	74,035
Prostate Cancer	Schumacher et al. 2018 Nat Genet	0.131	7.016	72,729
Prostate Cancer (UKBB)	UK Biobank	0.017	7.000	459,324
Breast Cancer (UKBB)	UK Biobank	0.018	6.846	459,324
Type 2 Diabetes	Morris et al., 2012 Nat Genet	0.095	6.463	60,786
Autism Spectrum	PGC Cross-Disorder Group, 2013 Lancet	0.622	6.191	10,263
Coronary Artery Disease	Schunkert et al., 2011 Nat Genet	0.075	6.016	77,210

Supplementary Table 2. Traits used in S-LDSC analyses. We report phenotype name, study reference, SNP heritability h_g^2, heritability Z-score, and number of samples N for the 63 independent diseases and complex traits (Gazal et al., 2021) used in S-LDSC analyses.

		CoalNN	ASMC mean posterior
**1st decile**	MAE	424 (2)	857 (6)
	RMSE	1151 (8)	2392 (17)
**2nd decile**	MAE	745 (7)	1164 (15)
	RMSE	1673 (20)	3200 (35)
**3rd decile**	MAE	1233 (19)	1683 (36)
	RMSE	2382 (34)	4070 (54)
**4th decile**	MAE	2465 (53)	3287 (83)
	RMSE	4193 (82)	6245 (106)
**5th decile**	MAE	4365 (67)	5696 (104)
	RMSE	6742 (99)	9163 (114)
**6th decile**	MAE	5927 (82)	7043 (96)
	RMSE	8743 (122)	10689 (122)
**7th decile**	MAE	7715 (72)	8060 (64)
	RMSE	10970 (101)	11859 (71)
**8th decile**	MAE	10741 (105)	9984 (92)
	RMSE	14286 (118)	13941 (104)
**9th decile**	MAE	15944 (150)	14452 (171)
	RMSE	20253 (179)	19063 (196)
**10th decile**	MAE	34508 (236)	33869 (284)
	RMSE	47456 (341)	47604 (422)

Supplementary Table 3. Accuracy of pairwise TMRCA prediction by decile of TMRCA distribution. We report the average performance of CoalNN and ASMC mean posterior in generations for the mean absolute error (MAE) and the root mean squared error (RMSE) for deciles of the true TMRCA distribution across 10 simulations of sequencing data. Numbers in round brackets represent standard errors.

Discretization bins		ASMC mean posterior	ASMC MAP	Runtime (mins)
**100**	MAE	8633 (34)	9676 (50)	180.60 (2.79)
	RMSE	18189 (158)	21410 (169)	
**200**	MAE	8594 (30)	9758 (45)	222.57 (2.81)
	RMSE	17975 (120)	21918 (126)	
**300**	MAE	8574 (28)	9822 (46)	282.77 (8.91)
	RMSE	17895 (105)	22285 (114)	
**400**	MAE	8562 (27)	9861 (45)	309.91 (2.52)
	RMSE	17853 (99)	22557 (109)	

Supplementary Table 4. Accuracy and runtime for different ASMC time discretizations. We report the average performance of ASMC for the mean absolute error (MAE) and the root mean squared error (RMSE) for different numbers of time discretization bins across 10 simulations for sequencing data. Runtime is the average number of minutes to decode all pairwise combinations of 200 haplotypes in batches of 100 pairs across the 10 simulations. Numbers in round brackets represent standard errors.

			CoalNN	ASMC mean posterior	ASMC MAP
Imputed	panel size = 300	MAE	10138 (79)	9996 (80)	11253 (80)
		RMSE	21094 (158)	20908 (167)	24265 (149)
	panel size = 1000	MAE	9234 (57)	9206 (54)	10482 (65)
		RMSE	19476 (132)	19433 (150)	23259 (139)
	panel size = 2000	MAE	8861 (84)	8911 (76)	10121 (84)
		RMSE	18689 (164)	18768 (161)	22600 (147)

Supplementary Table 5. Accuracy of pairwise TMRCA prediction in imputed data. We report the average performance in generations of CoalNN and ASMC for the mean absolute error (MAE) and the root mean squared error (RMSE) across 10 simulations. The reported performance is obtained by using the CoalNN sequencing weights and rounding the imputed allele dosages to binarize them. Numbers in round brackets represent standard errors. Reference panel sizes are in haploid units.

GC rate		CoalNN with GC	CoalNN without GC	ASMC mean posterior
0	MAE	NA (NA)	7982 (32)	8166 (27)
	RMSE	NA (NA)	13518 (73)	13262 (49)
1.0×10^{-8}	MAE	7901 (42)	8588 (27)	8897 (27)
	RMSE	13540 (72)	14610 (58)	14547 (61)
2.0×10^{-8}	MAE	8568 (22)	9141 (48)	9518 (51)
	RMSE	14767 (48)	15467 (64)	15484 (62)
3.0×10^{-8}	MAE	8881 (46)	9622 (30)	10000 (32)
	RMSE	15160 (67)	16178 (43)	16205 (47)
4.0×10^{-8}	MAE	9527 (32)	9978 (36)	10399 (36)
	RMSE	15958 (61)	16633 (59)	16699 (56)

Supplementary Table 6. Accuracy of pairwise TMRCA prediction for different non-crossover gene conversion rates. We report the average performance of CoalNN and ASMC mean posterior, in generations, using the mean absolute error (MAE) and the root mean squared error (RMSE). Simulations involve a constant effective population size of 10,000 diploid individuals, a constant recombination rate of 10^{-8} per base pair per generation, and different values of non-crossover gene conversion rate. Numbers in round brackets represent standard errors. Numbers in round brackets represent standard errors.

	AND gate	XOR gate
**Channel 1**	0.035 (0.001)	-0.009 (0.001)
**Channel 2**	0.024 (0.000)	-0.056 (0.001)
**Channel 3**	-0.008 (0.001)	0.017 (0.001)
**Channel 4**	0.014 (0.000)	0.002 (0.000)
**Channel 5**	-0.232 (0.001)	-0.016 (0.001)
**Channel 6**	-0.051 (0.000)	0.08 (0.001)
**Channel 7**	-0.272 (0.001)	-0.172 (0.001)
**Channel 8**	-0.057 (0.000)	0.101 (0.001)

Supplementary Table 7. Recovery of the AND/XOR features from raw haplotypes. Pearson correlation coefficient between the XOR/AND functions calculated on the raw haplotypes and the 8 channels of output of the first ConvBlock (see Methods). The numbers in brackets are standard errors calculated over 20 simulations.

DAF bin (%)	Number of dated variants					Median age (generations)				
	AFR	AMR	EAS	EUR	SAS	AFR	AMR	EAS	EUR	SAS
[0, 0.5]	5,156,014	2,248,935	1,819,869	1,648,497	2,159,787	2,160 (34)	1,570 (16)	1,045 (38)	960 (16)	927 (61)
[0.5, 1]	2,762,406	1,884,075	1,191,727	1,358,230	1,493,628	3,564 (53)	3,071 (53)	1,703 (25)	1,137 (9)	1,066 (16)
[1, 2.5]	3,082,203	1,923,613	913,021	1,181,402	1,384,546	4,904 (53)	4,852 (64)	2,634 (27)	1,593 (20)	1,517 (29)
[2.5, 5]	2,333,277	918,075	646,257	848,596	846,087	7,138 (65)	7,620 (67)	7,089 (93)	4,593 (62)	5,041 (47)
[5, 10]	2,028,081	958,302	736,868	904,633	930,768	1,0581 (71)	11,303 (82)	10,395 (79)	8,046 (48)	8,187 (48)
[10, 25]	2,350,274	1,536,898	1,321,044	1,479,233	1,547,460	17,193 (84)	17,737 (67)	13,441 (56)	12,138 (40)	11,802 (40)
[25, 50]	1,523,362	1,404,734	1,312,117	1,377,671	1,420,932	28,476 (95)	2,6709 (123)	17,970 (93)	18,847 (83)	17,873 (83)
[50, 100]	2,057,347	2,184,241	1,954,993	2,015,680	1,979,944	43,636 (153)	44,824 (201)	28,261 (198)	29,741 (150)	31,261 (172)

Supplementary Table 8. Median age of dated variants among different population groups. We report the number of variants dated by CoalNN and their median age in generations stratified by derived allele frequency (DAF) as observed within a given population group. We only considered non-singleton polymorphic variants for which high-confidence ancestral information was available (Paten et al., 2008). Numbers in round brackets represent standard errors obtained by bootstrapping (see Methods).

Derived allele frequency bin (%)	Number of dated variants			Median age (generations)		
	CEU	CHS	YRI	CEU	CHS	YRI
[0.5, 1]	N.A	516,114	1,477,617	N.A	628 (17)	3,818 (34)
[1, 2.5]	1,101,465	510,236	2,243,382	701 (18)	599 (17)	4,762 (33)
[2.5, 5]	44,8342	253,321	1,638,788	1,023 (20)	907 (30)	6,795 (34)
[5, 10]	225,484	147,593	1,374,079	2,203 (73)	2,165 (138)	9,438 (39)
[10, 25]	94,380	83,756	833,036	7,761 (314)	9,092 (418)	13,901 (57)
[25, 50]	7,934	12,096	118,731	13,850 (574)	17,104 (727)	22,201 (175)
[50, 100]	58,060	53,894	423,917	60,023 (719)	59,019 (493)	49,115 (215)

Supplementary Table 9. Median age of variants among different populations dated by CoalNN trained on a demographic prior of constant size. We report the number of variants dated by CoalNN trained on a constant population size ($N_e = 20,000$) and their median age in generations, stratified by derived allele frequency as observed within CEU, CHS or YRI. Only variants that remained polymorphic within each population were considered. Singletons were not included. Numbers in round brackets represent standard errors obtained by bootstrapping (see Methods). N.A denotes the absence of dated variant within the derived allele frequency bin for the corresponding population.

	DAF bin (%)	Number of dated variants					Median age (generations)				
		AFR	AMR	EAS	EUR	SAS	AFR	AMR	EAS	EUR	SAS
Benign	[0, 1]	60243	40505	36714	41811	21027	1083 (24)	812 (10)	741 (36)	524 (10)	510 (19)
	[1, 2.5]	7573	4742	2655	3384	3181	4187 (107)	3115 (142)	2311 (44)	1391 (21)	1416 (56)
	[2.5, 5]	5277	2217	1590	2185	2005	6364 (80)	5385 (313)	4807 (390)	2743 (126)	4004 (176)
	[5, 10]	4215	2098	1632	2028	2053	10116 (181)	9433 (312)	8917 (261)	6730 (242)	6868 (182)
	[10, 50]	7264	5876	5233	5724	5837	19695 (243)	19293 (329)	13908 (190)	13168 (188)	12956 (189)
	[50, 100]	3949	4367	3802	3997	3807	40953 (301)	41976 (444)	25168 (423)	27154 (441)	28143 (402)
Poss. damaging	[0, 1]	17193	11322	11085	13094	5926	949 (24)	720 (11)	709 (32)	510 (11)	480 (23)
	[1, 2.5]	1725	1100	685	829	794	3908 (116)	2204 (178)	2123 (70)	1284 (39)	1243 (34)
	[2.5, 5]	1042	480	351	510	439	5952 (244)	3812 (435)	4394 (505)	2255 (224)	3787 (457)
	[5, 10]	760	440	331	381	400	9055 (305)	7948 (412)	8509 (609)	6161 (390)	6096 (358)
	[10, 50]	1018	816	777	804	823	18243 (365)	17221 (578)	12037 (431)	11993 (421)	11295 (447)
	[50, 100]	130	174	211	172	174	38587 (970)	33168 (2346)	20898 (925)	22610 (1776)	24821 (1798)
Prob. damaging	[0, 1]	22284	15063	16221	19044	7941	846 (18)	675 (10)	677 (30)	511 (11)	445 (22)
	[1, 2.5]	1803	1145	696	975	878	3362 (135)	1719 (108)	2036 (65)	1226 (38)	1262 (49)
	[2.5, 5]	1067	425	375	440	419	5433 (179)	3390 (396)	3193 (230)	1816 (171)	2211 (289)
	[5, 10]	703	409	283	364	410	8963 (373)	6391 (538)	6673 (794)	5148 (468)	5190 (389)
	[10, 50]	810	694	678	694	654	17500 (444)	16570 (692)	12561 (420)	12095 (510)	11993 (369)
	[50, 100]	100	131	170	136	146	34742 (1125)	31530 (2180)	19270 (1157)	20549 (1538)	22579 (1657)

Supplementary Table 10. Median age of variants among different population groups annotated by PolyPhen-2. We report the number of annotated variants by PolyPhen-2 dated by CoalNN and their median age in generations, stratified by derived allele frequency (DAF) as observed within a given population group.

	DAF bin (%)	Number of dated variants					Median age (generations)				
		AFR	AMR	EAS	EUR	SAS	AFR	AMR	EAS	EUR	SAS
Tolerated	[0, 1]	63965	42999	39319	45297	22214	1059 (22)	797 (9)	733 (32)	523 (11)	505 (16)
	[1, 2.5]	7838	4929	2754	3578	3366	4113 (91)	3058 (140)	2284 (45)	1388 (26)	1407 (49)
	[2.5, 5]	5352	2275	1673	2263	2030	6335 (115)	5443 (323)	4453 (364)	2807 (123)	3952 (201)
	[5, 10]	4262	2169	1637	2049	2151	9946 (159)	9236 (264)	8912 (271)	6677 (269)	6689 (212)
	[10, 50]	7290	5859	5314	5723	5870	19505 (197)	19150 (328)	13741 (157)	13042 (169)	12804 (167)
	[50, 100]	3900	4346	3772	3958	3777	40922 (324)	41934 (463)	25187 (489)	27256 (442)	28157 (402)
Deleterious	[0, 1]	36868	24710	25536	29613	13149	900 (18)	698 (9)	698 (34)	510 (10)	467 (22)
	[1, 2.5]	3361	2121	1341	1685	1559	3716 (112)	1925 (91)	2154 (47)	1247 (29)	1222 (33)
	[2.5, 5]	2127	870	678	912	859	5761 (155)	3489 (259)	3954 (325)	1890 (128)	2923 (184)
	[5, 10]	1438	823	634	759	758	9243 (253)	7474 (425)	7638 (525)	5570 (360)	6267 (333)
	[10, 50]	1878	1563	1414	1532	1481	18326 (332)	17349 (428)	12800 (318)	12449 (337)	12143 (262)
	[50, 100]	294	370	451	384	391	36896 (1064)	32819 (1539)	20601 (507)	21681 (889)	23579 (1261)

Supplementary Table 11. Median age of variants among different population groups annotated by SIFT. We report the number of variants annotated by SIFT dated by CoalNN and their median age in generations, stratified by derived allele frequency (DAF) as observed within a given population group.

**Annotation**	**Effect size τ^* not including CoalNN**	**Effect size τ^* including CoalNN**				
		AFR	AMR	EAS	EUR	SAS
CoalNN	N.A	-0.219 (0.019)	-0.174 (0.019)	-0.184 (0.019)	-0.148 (0.018)	-0.187 (0.019)
LLD-AFR	-0.271 (0.004)	-0.267 (0.004)	-0.269 (0.004)	-0.268 (0.004)	-0.269 (0.004)	-0.268 (0.004)
Recombination rate	-0.370 (0.006)	-0.367 (0.006)	-0.369 (0.006)	-0.369 (0.006)	-0.369 (0.006)	-0.368 (0.006)
Nucleotide diversity	-0.051 (0.005)	-0.046 (0.005)	-0.050 (0.005)	-0.048 (0.005)	-0.050 (0.005)	-0.049 (0.005)
B-statistic	0.090 (0.004)	0.089 (0.004)	0.089 (0.004)	0.089 (0.004)	0.089 (0.004)	0.089 (0.004)
CpG content	0.321 (0.006)	0.321 (0.006)	0.321 (0.006)	0.321 (0.006)	0.321 (0.006)	0.321 (0.006)
ASMC_{avg}	-0.294 (0.004)	-0.292 (0.004)	-0.292 (0.004)	-0.291 (0.004)	-0.293 (0.004)	-0.292 (0.004)

Supplementary Table 12. S-LDSC analysis of CoalNN annotation on population groups. τ^* estimates (meta-analysed across 63 independent diseases and complex traits) of CoalNN MAF-adjusted allele age annotation on each population group (AFR, AMR, EAS, EUR and SAS) in independent marginal S-LDSC analyses conditioned on 96 baseline annotations (the full baseline model except for ARGweaver). We also report effect sizes of Baseline-LD evolutionary annotations (level of LD measured in African populations LLD-AFR, recombination rate, nucleotide diversity, B-statistic (McVicker et al., 2009), CpG content (Zhang et al., 2021) and average pairwise TMRCA ASMC_{avg} (Palamara et al., 2018)), before and after the introduction of the allele age annotation.

	ARGweaver allele age	ASMC_{avg}	B-statistic	CpG content	LLD-AFR	Nucleotide diversity	Recombination rate
ACB	0.64462	0.28373	-0.0449627	-0.0068927	0.357883	0.193833	-0.0115892
ASW	0.637648	0.262087	-0.0511699	0.0073261	0.308689	0.175725	0.0188068
BEB	0.566617	0.25335	-0.0249983	-0.0177649	0.315825	0.139136	-0.0245286
CDX	0.461265	0.273944	-0.0317672	-0.0211324	0.248805	0.143154	-0.0212694
CEU	0.599494	0.321749	-0.0459838	-0.0102032	0.314191	0.168587	-0.0112538
CHB	0.532193	0.31198	-0.0411218	-0.0324496	0.291585	0.166983	-0.0367068
CHS	0.462519	0.28222	-0.0184473	-0.0334043	0.331941	0.183432	-0.0848482
CLM	0.624238	0.29408	-0.0219222	-0.0232567	0.379901	0.181055	-0.0551782
ESN	0.545247	0.268409	-0.0438037	0.00498154	0.253668	0.187784	0.0165792
FIN	0.565649	0.294069	-0.00533878	-0.0418206	0.38372	0.143829	-0.0882545
GBR	0.597827	0.302148	-0.0403849	-0.00995346	0.308844	0.151967	-0.0144756
GIH	0.527073	0.244278	0.0185592	-0.0524937	0.405602	0.129283	-0.0885153
GWD	0.58232	0.260541	-0.0263054	0.000694733	0.2974	0.174894	-0.0206123
IBS	0.519219	0.276006	-0.0430249	-0.000972771	0.252918	0.12374	0.0289131
ITU	0.585557	0.305872	-0.0515937	-0.0102396	0.311108	0.182995	0.00317832
JPT	0.489656	0.282032	-0.0326494	-0.026941	0.275463	0.158177	-0.0352778
KHV	0.507532	0.253314	-0.0239953	-0.0244129	0.267331	0.125592	-0.0289965
LWK	0.584643	0.260102	-0.0550596	0.0119386	0.235916	0.178538	0.0261056
MSL	0.539203	0.243146	-0.0455326	0.0163128	0.214835	0.16732	0.0288875
MXL	0.59563	0.2825	-0.0410389	-0.0051117	0.312468	0.158115	0.000506131
PEL	0.59611	0.275924	-0.0391656	-0.00776521	0.314023	0.15596	-0.00922475
PJL	0.580811	0.293767	-0.057732	0.00708515	0.268913	0.167449	0.0398443
PUR	0.615749	0.274605	-0.0522987	0.0173573	0.283352	0.172801	0.0367664
STU	0.585506	0.265688	-0.0169196	-0.0237441	0.334048	0.159808	-0.0349042
TSI	0.592384	0.303182	-0.0535334	-0.0155446	0.296478	0.154585	0.00063352
YRI	0.550575	0.262261	-0.0448539	0.00265146	0.254752	0.185644	0.00491168

Supplementary Table 13. Correlation between MAF-stratified CoalNN allele age annotation and other evolutionary annotations. We report correlations computed on common SNPs (MAF $\geq$ 5%) between each of the 26 population specific MAF-adjusted CoalNN annotations and evolutionary annotations from the Baseline-LD model. ARGweaver allele age, ASMC_{avg}, and LLD-AFR annotations were also MAF-stratified.

a

**Annotation**	**Effect size τ^* not including CoalNN**	**Effect size τ^* including CoalNN**
CoalNN	N.A	-0,218 (0,018)
LLD-AFR	-0,271 (0,004)	-0,268 (0,004)
Recombination rate	-0,370 (0,006)	-0,368 (0,006)
Nucleotide diversity	-0,051 (0,005)	-0,048 (0,005)
B-statistic	0,090 (0,004)	0,089 (0,004)
CpG content	0,321 (0,006)	0,321 (0,006)
$ASMC_{avg}$	-0,294 (0,004)	-0,291 (0,004)

b

**Annotation**	**Effect size τ^* not including ARGweaver**	**Effect size τ^* including ARGweaver**
ARGweaver	N.A	-0.147 (0.005)
LLD-AFR	-0.271 (0.004)	-0.241 (0.004)
Recombination rate	-0.370 (0.006)	-0.348 (0.006)
Nucleotide diversity	-0.051 (0.005)	-0.039 (0.005)
B-statistic	0.090 (0.004)	0.083 (0.004)
CpG content	0.321 (0.006)	0.314 (0.006)
$ASMC_{avg}$	-0.294 (0.004)	-0.249 (0.004)

Supplementary Table 14. S-LDSC analysis of predicted allele age annotations. Numerical values from Fig. 5b. Effect size τ^* estimates (meta-analysed across 63 independent diseases and traits) of CoalNN MAF-adjusted allele age annotation on all 26 populations (in **a**) and of ARGweaver MAF-adjusted allele age annotation (in **b**), in marginal S-LDSC analysis conditioned on 96 baseline annotations (the full Baseline-LD model except for ARGweaver). We also report effect sizes of Baseline-LD evolutionary annotations (level of LD measured in African populations LLD-AFR, recombination rate, nucleotide diversity, *B*-statistic (McVicker et al., 2009), CpG content (Zhang et al., 2021) and average pairwise TMRCA $ASMC_{avg}$ (Palamara et al., 2018)), before and after the introduction of the allele age annotation.

Supplementary References

Adzhubei, I. A., Schmidt, S., Peshkin, L., Ramensky, V. E., Gerasimova, A., Bork, P., Kondrashov, A. S., and Sunyaev, S. R. (2010). A method and server for predicting damaging missense mutations. *Nature methods*, 7(4):248–249.

Byrska-Bishop, M., Evani, U. S., Zhao, X., Basile, A. O., Abel, H. J., Regier, A. A., Corvelo, A., Clarke, W. E., Musunuri, R., Nagulapalli, K., et al. (2022). High-coverage whole-genome sequencing of the expanded 1000 genomes project cohort including 602 trios. *Cell*, 185(18):3426–3440.

Gazal, S., Weissbrod, O., Hormozdiari, F., Dey, K., Nasser, J., Jagadeesh, K., Weiner, D., Shi, H., Fulco, C., O'Connor, L., et al. (2021). Combining snp-to-gene linking strategies to pinpoint disease genes and assess disease omnigenicity. *medRxiv*.

McVicker, G., Gordon, D., Davis, C., and Green, P. (2009). Widespread genomic signatures of natural selection in hominid evolution. *PLoS genetics*, 5(5):e1000471.

Palamara, P. F., Terhorst, J., S. Song, Y., and L. Price, A. (2018). High-throughput inference of pairwise coalescence times identifies signals of selection and enriched disease heritability. *Nature Genetics*, 50.

Paten, B., Herrero, J., Beal, K., Fitzgerald, S., and Birney, E. (2008). Enredo and pecan: genome-wide mammalian consistency-based multiple alignment with paralogs. *Genome research*, 18(11):1814–1828.

Shrikumar, A., Greenside, P., Shcherbina, A., and Kundaje, A. (2016). Not just a black box: Learning important features through propagating activation differences. *arXiv preprint arXiv:1605.01713*.

Sim, N.-L., Kumar, P., Hu, J., Henikoff, S., Schneider, G., and Ng, P. C. (2012). Sift web server: predicting effects of amino acid substitutions on proteins. *Nucleic acids research*, 40(W1):W452–W457.

Simonyan, K., Vedaldi, A., and Zisserman, A. (2014). Deep inside convolutional networks: Visualising image classification models and saliency maps. In *Workshop at International Conference on Learning Representations*.

Spence, J. P. and Song, Y. S. (2019). Inference and analysis of population-specific fine-scale recombination maps across 26 diverse human populations. *Science Advances*, 5(10):eaaw9206.

Terhorst, J., Kamm, J. A., and Song, Y. S. (2017). Robust and scalable inference of population history from hundreds of unphased whole genomes. *Nature Genetics*, 49(2):303–309.

Yu, F. and Koltun, V. (2015). Multi-scale context aggregation by dilated convolutions. *arXiv preprint arXiv:1511.07122*.

Zeiler, M. D. and Fergus, R. (2014). Visualizing and understanding convolutional networks. In Fleet, D., Pajdla, T., Schiele, B., and Tuytelaars, T., editors, *Computer Vision – ECCV 2014*, pages 818–833, Cham. Springer International Publishing.

Zhang, L., Dai, Z., Yu, J., and Xiao, M. (2021). Cpg-island-based annotation and analysis of human housekeeping genes. *Briefings in bioinformatics*, 22(1):515–525.
